# Beyond Color Boundaries: Pioneering Developments in Cholesteric Liquid Crystal Photonic Actuators

**DOI:** 10.3390/mi15060808

**Published:** 2024-06-20

**Authors:** Jinying Zhang, Yexiaotong Zhang, Jiaxing Yang, Xinye Wang

**Affiliations:** 1 Beijing Key Laboratory for Precision Optoelectronic Measurement Instrument and Technology, School of Optics and Photonics, Beijing Institute of Technology, Beijing 100081, China; 3120220661@bit.edu.cn (Y.Z.); 3120220654@bit.edu.cn (J.Y.); 3120205353@bit.edu.cn (X.W.); 2 Yangtze Delta Region Academy of Beijing Institute of Technology, Jiaxing 314001, China

**Keywords:** cholesteric liquid crystal, soft actuator, structural color camouflage

## Abstract

Creatures in nature make extensive use of structural color adaptive camouflage to survive. Cholesteric liquid crystals, with nanostructures similar to those of natural organisms, can be combined with actuators to produce bright structural colors in response to a wide range of stimuli. Structural colors modulated by nano-helical structures can continuously and selectively reflect specific wavelengths of light, breaking the limit of colors recognizable by the human eye. In this review, the current state of research on cholesteric liquid crystal photonic actuators and their technological applications is presented. First, the basic concepts of cholesteric liquid crystals and their nanostructural modulation are outlined. Then, the cholesteric liquid crystal photonic actuators responding to different stimuli (mechanical, thermal, electrical, light, humidity, magnetic, pneumatic) are presented. This review describes the practical applications of cholesteric liquid crystal photonic actuators and summarizes the prospects for the development of these advanced structures as well as the challenges and their promising applications.

## 1. Introduction

**Figure 1:**
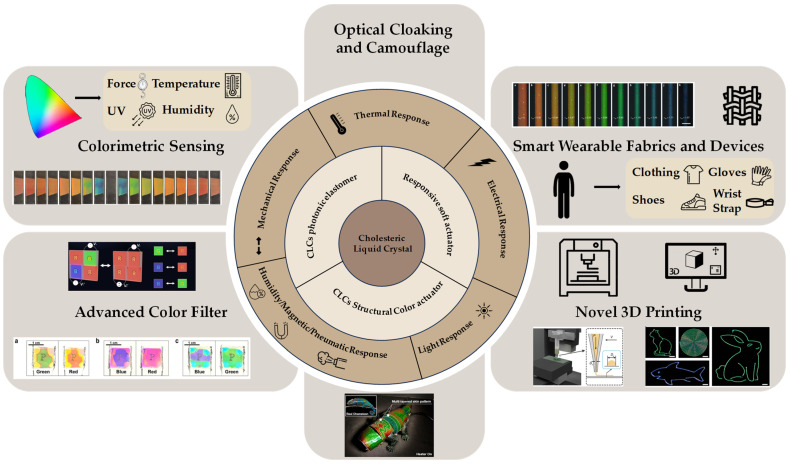
An overview of the classification, stimulus response, and potential applications of cholesteric liquid crystal photonic actuators [10,11,12,13].

Camouflage plays a vital role in the survival and thriving of organisms in nature. Organisms frequently benefit from survival advantages by blending into their natural surroundings or mimicking dangerous species, leading natural enemies to make erroneous judgments [1,2,3]. Biological camouflage specifically refers to the capacity of organisms to alter their color and texture, allowing them to adapt their camouflage in various environments, thus enhancing their survival flexibility. Camouflage in nature can be categorized as morpho-mimetic and color-mimetic. Morphological mimicry entails imitating the shapes and textures of surrounding objects to blend the biological appearance with the environment. Color mimicry represents another significant form of camouflage, wherein organisms achieve a visual effect of blending with the surrounding background by adopting colors and patterns similar to those of their environment. These camouflage strategies are widespread, not only observed among reptiles and arthropods but also extensively developed in fish and cephalopods. Swiss researchers were the first to discover the mechanism of color change that produces structural color in chameleons [4]. The skin of chameleons comprises two layers of densely overlapping cells: the upper layer contains tightly packed guanine nanocrystals, while the lower layer consists of larger, disorganized guanine nanocrystals. By altering the arrangement structure of these nanocrystals, chameleons can change their color. Upon light passage, these cells selectively reflect short wavelengths of blue light. When chameleons are tense, they actively adjust the sparseness of the crystals, causing their arrangement to become looser and reflecting light of longer wavelengths. As the gaps increase, the color changes from blue to green, yellow, orange, and red. These structured light color changes, generated using nanostructures on the surface of living organisms, represent dynamic adaptive camouflage. Likewise, Phelsuma lizards [5], squid [6], neon fish [7], and the chameleon shawfish Hoplolatilus chlupatyi [8] can achieve camouflage using similar nanostructures. Inspired by these organisms [9], liquid-crystal-based structural color soft actuators combine the deformation characteristics of soft materials and artificial muscles with real-time dynamic broadband color changes in the visible light range. They meet the requirements of both morphology mimicry and color mimicry, pushing the color boundaries of flat visuals and offering significant application prospects in the field of camouflage. In this review, we present the current state of research on cholesteric liquid crystal photonic actuators (CLCPAs) and their technological applications. As shown in Figure 1, an overview of the classification, stimulus response, and potential applications of CLCPAs is presented. First, we outline the basic concepts of cholesteric liquid crystals and their nanostructural modulation. And we present the latest progress in cholesteric liquid crystal photonic actuators responsive to various stimuli, including mechanical, thermal, electrical, light, humidity, magnetic, and pneumatic influences. Additionally, this review discusses the practical applications of cholesteric liquid crystal photonic actuators, summarizes the prospects for developing these advanced structures, highlights the associated challenges, and explores their promising applications.

## 2. Basic Concepts

### 2.1. Cholesteric Liquid Crystals

**Figure 2:**
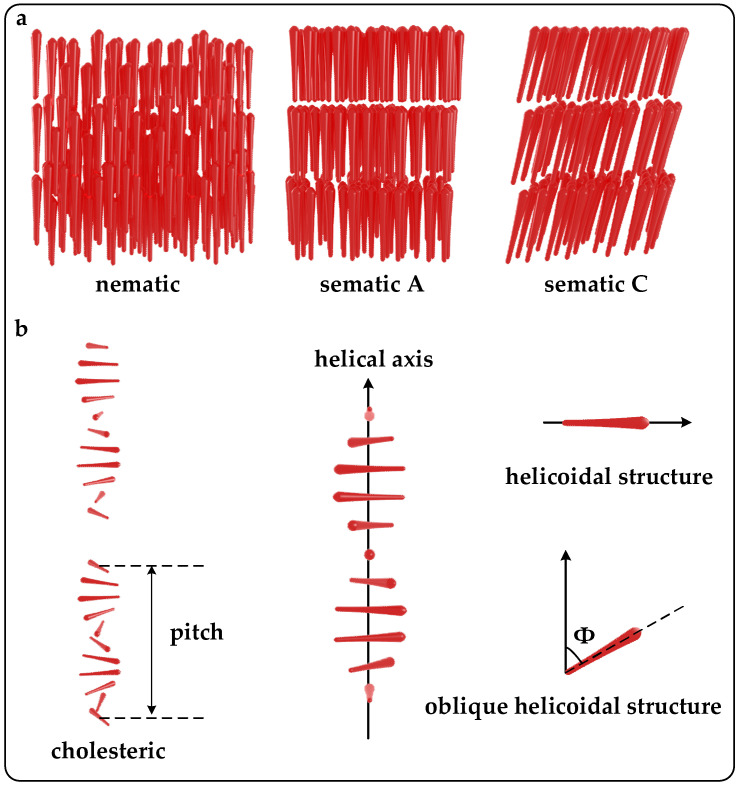
Illustration of LC molecules, alignment, and chiral pitch. (**a**) LC molecular arrangement of nematic, sematic A, and sematic C. (**b**) The arrangement of CLC molecules (where one molecule represents the molecular arrangement direction of the entire layer) and the pitch of CLCs are shown here as two cycles. To visually display the number of cycles, a virtual interval space is added. And two types of CLC nanostructures: helicoidal structure and oblique helicoidal structure.

Liquid crystals (LCs) constitute a distinct state of matter, exhibiting properties characteristic of both liquids and crystalline solids [14]. They possess anisotropic properties, wherein their physical characteristics vary depending on the direction of measurement. LCs can be classified into several types based on their molecular organization, including nematic, smectic, and cholesteric phases (Figure 2a). Cholesteric liquid crystals (CLCs), also known as chiral nematic liquid crystals, constitute a subclass of LCs distinguished by a helical molecular arrangement [15]. This helical structure imbues CLCs with unique optical properties, notably selective reflection of circularly polarized light (CPL). Depending on the pitch of the helix, CLCs can display vibrant structural colors that vary in response to external stimuli such as temperature, pressure, or electric fields.

In cholesteric liquid crystals, rod-like molecules arrange themselves into layers, and molecules in each layer are aligned parallel to one another in a specific direction and referred to as alignment directors. The molecules between two neighboring layers rotate at a certain angle, forming a periodic helical nanostructure. This rotation creates a spiral-like pattern along the axis of the helix. As this pattern repeats along the helical axis, it defines the pitch of CLCs, which represents the distance between layers in one complete cycle (alignment directors rotate 360°) of the helix (Figure 2b) [16]. The oblique helicoidal state of cholesterol refers to each layer’s director forming an angle with the helical axis, and this state can be modulated by electric, heat, magnetic, or light stimuli [17].

The pitch size ranges from tens of nanometers to a few micrometers. CLCs have single helical nanostructures that can be regarded as one-dimensional photonic crystals. When waves are incident on a periodic structure with the pitch, they are reflected from different planes within the structure. When the path difference between these reflected waves is an integer multiple of the wavelength, constructive interference occurs, resulting in strong reflected waves. This condition is described by Bragg’s Law [18,19]. Thus, if the wavelength of light aligns with the optical range of the pitch (pitch multiplied by the average refractive index), incident light on CLCs will be reflected, in accordance with Bragg’s law [20,21]:

(1) λ=n×p×cosθ

where λ is the center wavelength of selective reflection, n is the average refractive index, p is the pitch of the helical photonic nanostructure, and θ is the angle of incidence.

At the normal incidence, the selective reflection bandwidth BW is given by $BW=(n_{e}-n_{o})p=∆np$, where $n_{o}$ and $n_{e}$ represent the ordinary and extraordinary refractive indices, respectively [22].

The above equation describes the relationship between the reflected center wavelength and pitch in CLCs. Helical nanostructures are typically characterized by two parameters, namely helical pitch (p) and chirality [23]. As the orientation of the CLC helical structures does not overlap with its mirror image, it is inherently chiral. This nano-photonic structure can exhibit either left- or right-handedness, depending on the type of chiral agent. Therefore, a CLC cannot reflect more than 50% of normally incident unpolarized light. When the incident light beam is circularly polarized with opposite handedness to that of the CLC, it reflects 0% [24]. Utilizing a multilayer system or employing wash-out/refill techniques can surpass this limit, increasing the reflectance of unpolarized incident light to typically above 50%. The use of chiral liquid crystals with thermally induced inversion of helicity has also been demonstrated to achieve this [25]. By adjusting the type and mass ratio of the chiral agent, the pitch (p) as well as the chirality of the helical photonic structure can be adjusted, as given by the following equation [26]:

(2) $$p=\frac{1}{c\times HTP},$$

where c is the doping concentration of the chiral agent in the system, and helical twisting power (HTP) describes the magnitude of the spinning ability of the chiral dopant for a particular liquid crystal receptor; however, if the same chiral agent is added to different liquid crystal receptors, its spinning ability varies. With an increase in the concentration of the chiral dopant, the spinning capability in the system increases, leading to a decrease in CLC pitch and a blue shift in the initial color.

### 2.2. CLC Structural Color Elastomers

(a) CLC structural color elastomers

Structural color elastomers are responsive materials composed of polymers that can be triggered by an external stimulus to undergo shape and structural color changes either simultaneously or sequentially. In contrast to CLCs in cartridges, cholesteric liquid crystal elastomers (CLCEs) formed through crosslinking are small, lightweight, and capable of responding to multiple stimuli. Depending on the applied mechanical force, these elastomers can dynamically change both shape and structural color. When the elastomers are stretched and their length elongates, their thickness decreases, leading to a decrease in pitch and a shift of the reflected light towards shorter wavelengths. Conversely, when we release the stretch and allow the elastomers to contract, the pitch increases, causing the reflected light to shift towards longer wavelengths until it returns to its initial color. This process can be described by the following equation:

(3) $$∆\lambda =n\times p=n(p_{0}-p_{1})=n(p_{0}-p_{0}\frac{d_{1}}{d_{0}}),$$

where ∆λ is the shift of the selective reflection center wavelength, $p_{0}$ is the initial pitch when the elastomers are not stretched, $p_{1}$ is the changed pitch, $d_{0}$ is the initial thickness, and $d_{1}$ is the changed thickness. This equation essentially establishes a link between the selective reflection wavelength shift and the elastomer thickness change. This unique feature makes them highly adaptable for a wide range of applications where multifunctionality and responsiveness are desired.

CLC structural color elastomers stand at the forefront of material engineering, offering a blend of compactness and lightweight design that surpasses traditional boundaries. Within the elastic limits of an object, stress is directly proportional to strain, with their ratio defined as the Young’s modulus of the material. The magnitude of the Young’s modulus reflects the rigidity of the material; a higher Young’s modulus indicates lower susceptibility to deformation. Controlling the crosslinking density can alter the Young’s modulus of an elastomer, thereby facilitating pixelated control over its structural color. For CLC elastomers, the Young’s modulus can be expressed as follows [27,28]:

(4) $$E=-\frac{\sigma_{t}}{\varepsilon_{t}}=\frac{F/A}{∆L/L_{0}}=\frac{F/A}{(L_{0}-L)/L_{0}},$$

where E is the Young’s modulus of the soft actuator, $\sigma_{t}$ is the tensile stress, $\varepsilon_{t}$ is the tensile strain, F is the mechanical force stimuli, A is the cross-section area, ∆L is the amount of change in length, $L_{0}$ is the initial length, and L is the length after stretching.

To quantify the change in thickness during elastomer deformation, which subsequently influences the helical nanostructure, Poisson’s ratio was introduced. Poisson’s ratio is defined as the ratio of the absolute value of the transverse strain to the axial strain when the material is under tension. When subjected to one- or two-dimensional stretching in the plane, the strain in the thickness direction is related to Poisson’s ratio as follows:

(5) $$\nu =-\frac{\varepsilon_{d}}{\varepsilon_{t}}=\frac{d_{0}-d_{1}}{d_{0}\cdot \varepsilon_{t}},$$

where ν is Poisson’s ratio, and $\varepsilon_{d}$ is the strain in the thickness direction. Combining Equations (3)–(5), the following relationship is obtained:

(6) $$∆\lambda =n\cdot ∆p=n\cdot p_{0}\cdot \nu \cdot \varepsilon_{t}=\frac{n\cdot p_{0}\cdot \nu \cdot \sigma_{t}}{E},$$

For structural color soft actuators, it indicates that the offset of the reflection center wavelength is inversely proportional to the Young’s modulus.

(b) CLC structural color fibers

CLC structural color fibers have been intensively developed for smart fabrics. For CLC structured color fibers, due to the special shape of the cylinder, the calculation is different from that for normal CLCEs. In the context of tensile strain calculations, it is essential to account for the three-dimensional structure of CLC structural color fibers due to their cylindrical shape, unlike the planar configuration of conventional CLCEs. The cylindrical shape of the fiber leads to complex deformations in three-dimensional space under tensile strain, significantly influencing its optical properties. Therefore, it is crucial to accurately incorporate the fiber’s three-dimensional geometry into the model and utilize appropriate mathematical methods to describe the relationship between deformation and optical properties. Taking into account the response of such fiber to mechanical force, we can obtain the following relationship [11,29]:

(7) $${\left(1+\varepsilon_{z}\right)}^{-\nu_{f}}=(1+\varepsilon_{r})=(1+\frac{∆r}{r_{0}})=\frac{r}{r_{0}}=\frac{p}{p_{0}}=\frac{\lambda }{\lambda_{0}},$$

where $\nu_{f}$ is Poisson’s ratio of the fiber, r denotes the radius of the fiber after stretching, $r_{0}$ denotes the initial radius, $\varepsilon_{r}$ is the strain in the radial direction along the cylinder, and $\varepsilon_{z}$ is the strain in the axial direction z. This equation explains that for CLCE cylindrical fibers, as stress is applied, at the macroscopic level, both normal and radial strains occur simultaneously, causing a reduction in fiber radius. At the microscopic level, the pitch of the nano-helical structure decreases, leading to a blue shift in the reflected structural color spectrum as the center wavelength moves towards shorter wavelengths. According to Equation (7), we can obtain

(8) $$\lambda =\lambda_{0}{\left(1+\varepsilon_{z}\right)}^{-\nu_{f}},$$

The relative wavelength shift ∆λ is defined as

(9) $$\begin{array}{c} ∆\lambda =\frac{\lambda_{0}-\lambda }{\lambda_{0}}=\frac{\lambda_{0}(1-{\left(1+\varepsilon_{z}\right)}^{-\nu_{f}})}{\lambda_{0}}=1-{\left(1+\varepsilon_{z}\right)}^{-\nu_{f}} \end{array}$$

This shows that when CLC structural color fibers are subjected to stretching, their reflected color is related to the strain as well as the Poisson’s ratio. In the same material, we assume that Poisson’s ratio is constant, and then the greater the strain, the greater the wavelength shift of the reflection.

(c) Advantages of CLC structural color elastomers

Now we have covered the law of structural color change of CLCEs under deformation conditions. CLCEs enable the fabrication of structural color patterns with a wide range of customizable shapes at significantly lower costs compared to conventional LCDs. This innovative approach not only addresses the growing demands of modern technology for miniaturization and portability but also explores new opportunities for diverse applications across various sectors. These elastomers provide a transformative solution, revolutionizing industries from healthcare to consumer electronics and from microdevices to portable gadgets. CLCEs epitomize rapid response capabilities, swiftly reacting to external stimuli to effect changes in shape and color, particularly in the field of camouflage. These elastomers provide real-time adjustability and feedback mechanisms, ensuring seamless integration into dynamic environments and meeting the evolving demands of advanced photonic camouflage.

### 2.3. CLC Structural Color Actuators

The combination of CLC structural color elastomers with flexible actuators opens up a lot of possibilities for achieving vibrant and dynamic stimulus-responsive structural colors. Integrating one or more layers of flexible actuators with CLCEs facilitates the attainment of a broad spectrum of actuation modes and response patterns (stretching, compression, bending, twisting, depression, etc.) [30]. The integration of flexible actuators enhances the versatility and functionality of CLC structural color elastomers by enabling precise control over shape, deformation, and response to external stimuli. This combination presents unprecedented opportunities for the development of adaptive and responsive materials and devices. The CLC structural color actuator has a highly flexible backbone with both the optical properties of an LC material and the rubbery properties of an elastomer. The CLC structural color actuator consists of a crosslinked LC backbone with main or side chains, which has the ability to withstand large mechanical deformations. By editing the CLC system, shape memory [31,32] and self-healing [33,34] can be accomplished by utilizing dynamic covalent bonding and other reactions.

CLC structural color elastomers and flexible actuators are multilayered for multiple stimulus responses. The deformation of the actuator involves various types, thereby exhibiting complex color-changing behavior. For example, changes in temperature can induce alterations in molecular alignment, resulting in shifts in both shape and color [10,35]. Likewise, exposure to different wavelengths of light can trigger distinct structural color changes through isomerization or photothermal reactions [36,37]. Additionally, fluctuations in humidity levels can cause the actuators to swell or contract, resulting in corresponding modifications in their overall shape and optical properties [38,39]. The application of magnetic fields can exert directional forces on the magnetic particles doped in elastomers, further influencing their structural configuration and color appearance [39]. Their specific syntheses and applications will be described in more detail below. This remarkable adaptability to a variety of external stimuli underscores the great potential of CLC structural color elastomers for a wide spectrum of applications, including dynamic displays [40,41], adaptive camouflage [42], and responsive sensors and actuators [43].

CLC-based adaptive planar optics have become a focal point of research due to their ability to respond to various external stimuli, such as electric fields, magnetic fields, heat, light, and mechanical stress. These stimuli induce dynamic changes in LC structures, enabling active optical functionalities. Therefore, understanding recent progress in stimuli-responsive CLCs is crucial for elucidating the mechanisms behind the dynamic manipulation of LC structures and their implications for active optics. In the following sections, we will delve into the intricacies of dynamically adjustable cholesteric liquid crystal actuators, examining their design principles, fabrication techniques, and emerging applications in adaptive optics and beyond. Through this exploration, we aim to offer a comprehensive overview of advancements in CLC-based adaptive optics and their potentials in applications.

## 3. Dynamically Adjustable Cholesteric Liquid Crystal Structural Color Actuators

CLC-based adaptive planar optics have gained considerable attention for their responsiveness to external stimuli, including mechanical stress [44,45,46], heat [10,47,48,49], electric fields [50,51], light [52,53], and magnetic fields [39,54]. Dynamic manipulation of LC structures is crucial for advancing active optical applications and developing more sophisticated optical functionalities. In the following paragraphs, we will explore recent progress in stimuli-responsive CLCs and their applications in active optics.

### 3.1. Mechanical Response CLC Photonic Actuators (CLCPAs)

**Figure 3:**
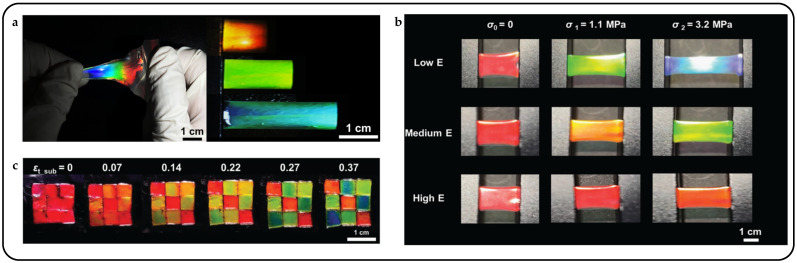
Mechanical response liquid crystal color actuator. (**a**) Mechanical stretching induces reflective color change in CLCPA samples [56]. (**b**) Structural color changes of CLCPA under stress with different elastic modulus [57]. (**c**) Observation of multicolor separation in a 3 × 3-pixel arrangement under biaxial strain, yielding corresponding macroscopic images [57].

Mechanical response is the most direct and straightforward method for actuating cholesteric liquid crystal actuators, particularly in complex multilayer structures such as sandwich or Janus structures [55]. Mechanical force stimulation typically results in large displacements and significant deformations and facilitates system integration, allowing CLCPAs to achieve continuous, tunable, and wide-range reflection peaks in the visible spectrum. Freestanding mechanical response CLCPAs can be obtained by perfusing CLC precursors into glass tunnel templates after UV polymerization [56]. With increasing stretching strain, a blue shift in the photonic wavelength was observed, from 695 nm to 494 nm, indicating a shift towards shorter wavelengths (Figure 3a). To simplify the modulation of reflective structural color in response to mechanical force stimuli, variable pixel units can be obtained on the same elastic substrate by designing various Young’s moduli in advance. A series of elastic modulus values were achieved by adjusting the crosslinkers and thiol chain extenders to alter the concentration of excess acrylate, thereby modifying the crosslink density (Figure 3b) [57]. CLCPAs with heterogeneous elastic moduli were simultaneously positioned on the same stretchable substrate and stretched, resulting in simultaneous color separation from a single color to multiple colors. The multicolor separation capability of the stretchable CLCEs was investigated under one- and two-dimensional stretching, and further development of CLCPAs was pursued for device applications (Figure 3c). Pixelated CLCs on a stretchable commercial substrate exhibit varying structural colors attributed to their designed elastic moduli. Additionally, this structure can be combined with other stimuli actuators to achieve pixelated structural color responses to thermal, electrical, optical, and other stimuli.

**Figure 4:**
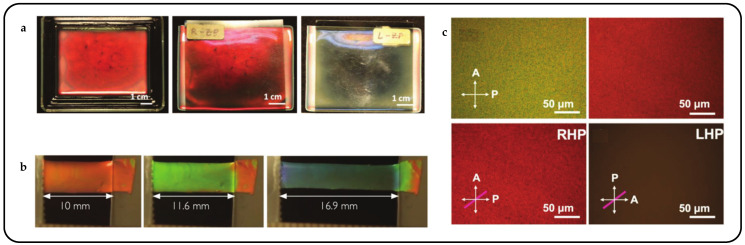
CLCE obtained by a method based on anisotropic deswelling. (**a**) CLCE with initial color red and its image through the left/right-hand polarizer [60]. (**b**) Structural color changes of CLCE under different stretching lengths [60]. (**c**) Polarizing optical microscopy (POM) images in transmission or reflection (with or without left/right-hand polarizer) [60].

Pixelated mechanical force-responsive CLCEs with real-time color change show great potential for development in next-generation displays. The preparation method for obtaining a flexible actuator with a uniform initial color over a large area is of practical importance. The “anisotropic deswelling” method, proposed by the Finkelmann group, was the first to achieve large areas of uniformly colored CLCEs [58]. This solution involved continuous centrifugation at elevated temperatures for 10 h. During the first half, the hydrosilation reaction occurred, followed by solvent evaporation in the second half. Although this method produced a visually significant improvement, and subsequent work reduced the centrifugation time to 5 to 8 h [59], the method was very cumbersome and needs further improvement. Rijeesh Kizhakidathazhath et al. presented a concise and refined method for fabricating large-area CLCEs with uniform coloration (Figure 4a) [60]. The method relied on a two-stage thiol-acrylate Michael addition and photopolymerization reaction to produce non-chiral nematic main-chain elastomers, transitioning to CLCEs through the introduction of the diacrylate chiral mesogen LC756. Additionally, this refinement allowed the process to proceed without centrifugation and at near-room temperature, while anisotropic deswelling was utilized to control helix orientation. The procedure entailed casting the CLC precursor solution onto a substrate, where it reacted to form a CLCE film exhibiting selective reflection color in the visible spectrum. When the film was stretched uniaxially or biaxially orthogonal to the helical axis, a notable blue shift occurred, promptly returning to the original state upon strain removal, even after prolonged strain application (Figure 4b). CLCs exhibited a distinction between left- and right-handed nano-helical axes, discernible by incorporating left- and right-handed polarizers into the optical path in the reflected state (Figure 4c). The nanostructure will reflect light that is identical to its chirality and transmit light that is opposite to its chirality. Leveraging this property, it becomes possible to manufacture one or more layers of optical filters with tunable selective reflection characteristics in terms of bandwidth and central wavelength.

In order to obtain self-healing in flexible materials, structures with specific functions were designed to be incorporated into polymer systems [61]. Common dynamic covalent bond exchange reactions of CLCPAs included transesterification [62], disulfide metathesis [63], diselenide metathesis [64], Diels–Alder reaction [65], and addition–fragmentation chain transfer [66]. As shown in Table 1, common dynamic covalent bond networks are summarized.

**Figure 5:**
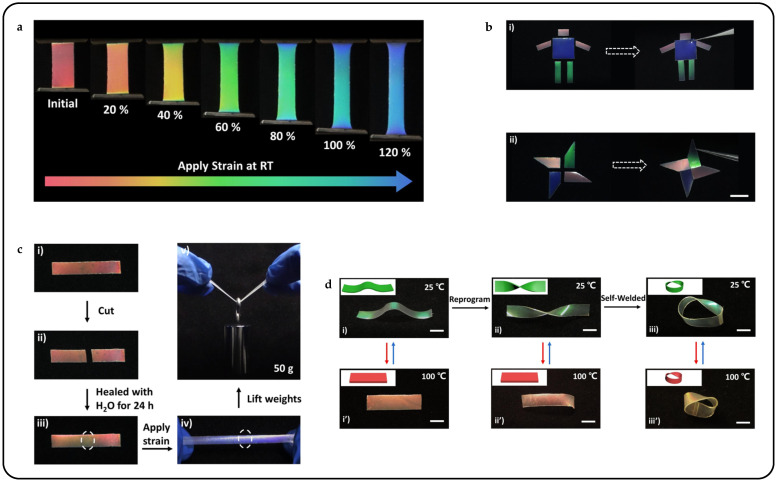
Shape-programmable, and self-healable CLCPA. (**a**) Stretching by mechanical force at room temperature [71]. (**b**) Self-healing demonstration of cartoon (**i**) and windmill (**ii**) patterns [71]. (**c**) Self-healing process of CLCPA with red initial color (**i**–**iii**) and its mechanical properties (**iv**,**v**) [71]. (**d**) Demonstration of motion through photonic actuator deformation driven by temperature difference [71]. Each actuator can reversibly change the 3D shape (**i**–**iii**, **i’**–**iii’**) and color (**i**–**i’**, **ii**–**ii’**, **iii**–**iii’**) at temperatures of 25 °C and 100 °C.

By integrating dynamic covalent boronic ester bonds into the main-chain CLCE polymer network, a CLCE material with shape-programmable and self-healable properties was successfully designed and synthesized [71]. Also based on anisotropic deswelling, shape-programmable CLCEs were achieved utilizing the thermo-activated boronic ester B-O bond exchange [78]. This reaction not only fixed the helicoidal orientation of mechanically aligned CLCEs but also facilitated the programming of their arbitrary colors and 3D shapes. Specifically, the research unveiled remarkable dynamic mechanochromic behaviors exhibited by CLCE films at room temperature. For example, during mechanical stretching, the red-reflecting CLCE film underwent a continuous color transition from red to blue, and the entire process was fully reversible, with no discernible delay observed between mechanical relaxation and color recovery (Figure 5a). Illustrated in Figure 5c, a red-reflecting CLCE film, when cut into two pieces, could be rejoined by applying a few drops of water across the damaged interface. Remarkably, the self-repaired sample demonstrated the ability to withstand a weight of 50 g, which was approximately 1000 times the weight of the film itself. Leveraging the self-healing properties of CLCEs, additional showcases featuring cartoon man and windmill patterns with various reflection colors were investigated (Figure 5b). When the actuators were subjected to cyclic heating and cooling between 100 °C and 25 °C, they demonstrated the capability not only to change colors but also to undergo reversible transformations between 3D shapes and 1D CLCE films (Figure 5d).

**Figure 6:**
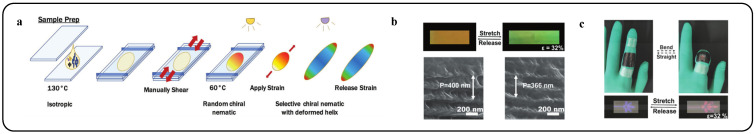
CLCPA with a mechanical force response to chain transfer. (**a**) Schematic of the polymerization and programming process [43]. (**b**) Structural color shift of multilayered CLCPA and its pitch changing by SEM [80]. (**c**) Knuckle flexion detector with flower pattern indication [80].

Shape programming and color change control of CLCPAs could also be achieved using covalent adaptable networks [79]. Alina M. Martinez et al. employed radical-mediated addition-fragmentation chain transfer (AFT) reactions to enable the permanent programming or erasure of thermoreversible shape and color by relieving stress within the strained network through reversible bond exchange [43]. During the actuator preparation stage, the synthesis of LC diacrylate oligomers was achieved through a thiol–Michael addition reaction, introducing AFT chemistry into the main network (Figure 6a). The CLCPA was designed with the latent capability for bond exchange with light-activated AFT, enabling network reconfiguration according to the spatiotemporal control provided by light activation.

Chain-transferred CLCPAs have demonstrated a broad spectrum of applications, including beetle bionics [81] and the development of brightly colored paints [82]. To enhance the actuation properties of CLCs, composite structural materials with multiple layers of elastomers are often required. Lifan Lu et al. presented a straightforward method for preparing CLCE films by incorporating a chain-transfer agent [80]. When sandwiched between two thermoplastic polyurethane (TPU) films, the resulting TPU/CLCE/TPU sandwich films exhibited mechanochromic behaviors (Figure 6b). This approach capitalized on the property of the chain-transfer agent, which effectively reduces the molecular weights and crosslinking degrees of polymers in the composite structure. These films held promise for applications in information storage and display in wearable devices. Figure 6c demonstrates a visually recognizable knuckle flexion detector utilizing a CLCPA with a sandwich structure.

**Figure 7:**
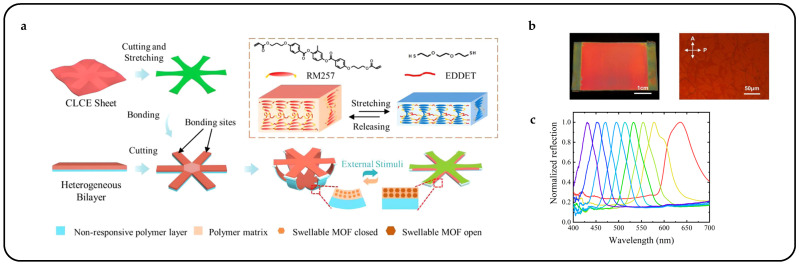
CLCPA for bionic flowers. (**a**) Stretching by mechanical force at room temperature [84]. (**b**) Photograph of a red-reflecting CLCE sheet and POM image of CLCPA in reflection mode with crossed linear polarizers [84]. (**c**) Normalized reflection spectra of the CLCPA sheet during stretching [84].

Multilayer structures play a crucial role in biomimetics, especially in emulating the complex structures found in biological organisms. This characteristic mirrors the regulatory abilities of biological organisms and their responsiveness to their environment, serving as a model for designing adaptive optical materials [83]. Using metal–organic framework (MOF)-based composite films as rigid actuation substrates enabled the support and mechanical guidance of spatial configurations for flexible CLCE sheets [84]. The components mainly consisted of two layers: the CLC sheet and the MOF-based actuation substrate (Figure 7a). Direct structural color programming of the CLCE sheet was facilitated by adjusting the thickness distribution, thus eliminating the need for cumbersome chemical modifications. Figure 7b depicts a red CLCPA photo along with a POM image. The deformation of the released flowers caused a shift in the CLCPA selective reflectance spectra, as depicted in Figure 7c.

A CLC fibroid is a type of raw material for smart textiles with intelligent responsive characteristics, enabling color changes or transparency adjustments based on external conditions [85]. Embedding CLCPAs into textiles enables smart temperature regulation to be achieved [86]. As the environmental temperature rises, alterations in the arrangement of liquid crystal molecules result in shifts in the fabric’s color or adjustments in transparency. CLC fibroids can also find applications in the field of intelligent health monitoring. By monitoring the wearer’s physiological parameters, such as biomolecule analysis [87], humidity [88], sweat [89], and pressure [90], smart textiles can dynamically adjust color or transparency, providing timely health alerts and monitoring functions. Additionally, liquid crystal smart textiles offer environmental benefits and energy savings by reducing energy consumption and waste generation, aligning with the concept of sustainable development.

**Figure 8:**
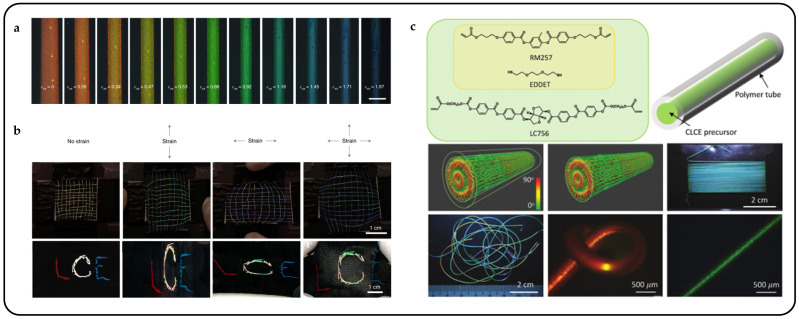
CLC structured color fibers with real-time response. (**a**) The reflection-mode POM images depict the initial red-reflection CLCE fiber under elongational strain, with a scale bar of 200 μm [11]. (**b**) The reactive liquid crystal monomer RM257 and chain extender EDDET react to form nematic liquid crystal oligomers with the chiral dopant LC756 added to induce a cholesteric phase. Large-scale views of uniaxial and biaxial mechanochromic responses under ambient light are observed in a simple weave of CLCE fibers and a single long CLC fiber sewn into a passive elastic cloth in the shape of a ‘C’ [11]. (**c**) Schematic representations illustrate the development of cholesteric order with radial helices within the tube and depict the state after crosslinking and tube removal. Macroscopic views display a 2.5 m long CLCE fiber with blue-green color collected on a flat winder, along with several fibers with red, and green colors respectively, randomly placed on a black table [29].

Recently, mechanochromic CLCE fibers were successfully fabricated using an innovative method [11]. An extruded oligomeric CLCE precursor solution was deposited onto a rotating mandrel and subsequently subjected to photo-crosslinking after annealing to form CLCE fibers (Figure 8a,b). However, this approach had inherent limitations: the fiber cross-section generated by the production method is strip-shaped rather than cylindrical. The subtle balance of the process occurring before crosslinking makes it sensitive to any deviation from the optimal processing parameters. Consequently, expanding core-based processes to meet industrial output expectations is challenging. To address these constraints, a novel strategy was devised wherein the CLCE fiber synthesis took place within the core of a low-density polyethylene tube, which could be readily dissolved after post-process completion [29]. This method not only facilitated the templating of cylindrical shapes and mitigated breakage due to capillary forces but also ensured confinement within the tube, thus promoting tangential alignment along the tube wall. This confinement promoted tangential alignment along the tube wall, enforcing the desired radial alignment as the cholesteric structure of the precursor liquid crystal developed within the fiber (Figure 8c).

### 3.2. Thermal Response CLC Photonic Actuators

Thermal response CLC actuators are intelligent material devices capable of changing their shape in response to heat stimuli [91]. These devices utilize CLCs as the base material, and they exhibit characteristics of shape change with temperature variation, making them suitable for precision control in mechanical devices at the nanoscale. Thermal stimulus-responsive CLC actuators find wide applications in the field of non-contact control. Controlling the temperature allows the precise adjustment of the helical structure of CLCs, enabling nanoscale control of structural color and morphology.

**Figure 9:**
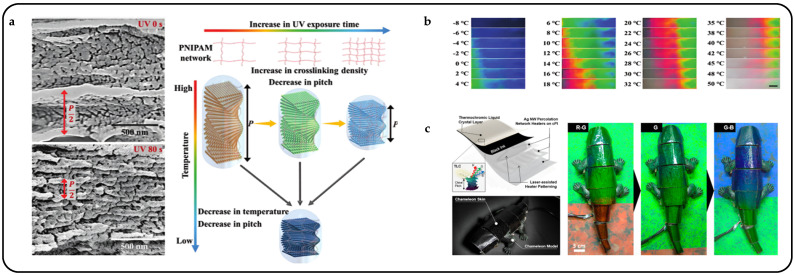
Thermal response CLC photonic actuators. (**a**) The scanning electron microscope (SEM) image depicts the CLC structure of the cholesteric phase cellulose composite (CPCC) [99]. (**b**) Temperature-responsive structural color photographs showcase the CPCC’s diverse response degrees to temperature variations [99]. (**c**) The arrangement and adaptive camouflage capabilities of a typical artificial chameleon robot [100].

Cellulose is now an emerging chiral liquid crystal spiking material that self-assembles nematic liquid crystals into cholesteric liquid crystals [92]. Cellulose nanocrystals play the role of a rigid backbone and have the same optical property of birefringence as liquid crystals. By incorporating cellulose nanocrystals into liquid crystal systems, CLCs with bright structural colors can be obtained after complete evaporation of the solvent [93]. It has been shown that cellulose nanocrystals exhibit left-handedness, selectively reflecting left-handed CPL and transmitting right-handed CPL [94], and that the resulting color can be altered by the addition of glucose [95] or sodium chloride [96] or by applying an electric [97] or magnetic field [98] to a suspension of cellulose nanocrystals to induce a change in pitch value. Xiaoxiang Wen et al. presented a methodological advancement in the fabrication of a thermochromic and circularly polarized cholesteric phase cellulose composite (CPCC) [99]. Drawing inspiration from the color-shifting and polarizing attributes observed in beetles, the CPCC is synthesized by combining hydroxypropyl cellulose with a cholesteric liquid crystal structure and a crosslinked PNIPAM network. Importantly, the density of crosslinking affects the thermochromic response, which can be finely adjusted by modulating the UV exposure duration during PNIPAM synthesis. As the temperature rises, the CPCC exhibits a red shift in color, indicating an expansion in the pitch of the CLC structure. As anticipated, when the temperature increases, the pitch increases, which causes a red shift in the reflected colors, as depicted in Figure 9a. With the increase in temperature, the color of CPCC experiences a redshift, indicating an expansion in the pitch of the CLC structure in response to the rising temperature (Figure 9b).

Recently, CLCs featuring intra-mesogenic supramolecular bonds were synthesized to enhance tunability and accelerate the response rate three-fold [101]. These materials incorporate LC monomers derived from dimerized oxy-benzoic acid (OBA) derivatives. Increasing the concentration of OBA comonomers amplifies the red-shifting thermochromism of selective reflection in the CLCPAs. Beyond a critical concentration threshold, selective reflection in the CLCPAs can be extinguished upon heating, akin to an on-off “switching” behavior. At room temperature, intact OBA groups dimerize via hydrogen bonding, forming a liquid crystalline diacrylate, resembling conventional LC monomers. High concentrations of 6-OBA lead to cleavage, reducing birefringence and enabling reversible clearing of selective reflection. The shape memory properties enabled by hydrogen bonding mesogens preserve deformed helical architectures, amplifying the thermochromic response.

Thermally responsive CLCPAs can be precisely controlled by Joule heat for deformation and structural color adaptive camouflage. Hyeonseok Kim et al. introduced a chameleon-inspired robot designed to actively sense its surroundings and dynamically adjust its surface coloration to seamlessly blend with the environment, achieving an advanced level of artificial camouflage [100] (Figure 9c). The robot integrates color adaptation through a closed-loop feedback system comprising a color sensor, a power controller based on proportional–integral–derivative (PID) control, and an artificial chameleon skin. The skin serves as an active color-displaying component, comprising a multilayer flexible film with patterned Ag nanowire heaters, black absorption ink, and thermochromic LC ink. The LC ink used is a commercially available cholesteric liquid crystal, which remains transparent at temperatures below 20 °C. Thus, in its native state, the CLCPA exclusively displays the inherent color of the black ink. Precise and independent temperature control can be achieved over each heater by individually connecting the input nodes of different heater layers to external electrodes, allowing distinct patterns to be transferred onto the CLC layer. As a result, diverse colors can be manifested in various patterns within the CLCPA.

**Figure 10:**
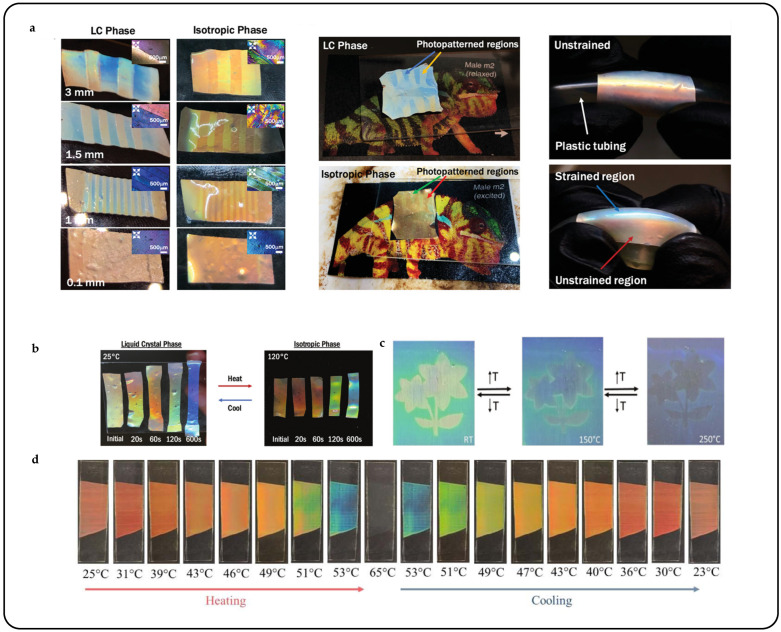
Patterned camouflage of thermal response CLCPA. (**a**) The images depict the CLCPA material at two different temperatures: at room temperature in the LC phase and 120 °C in the isotropic phase. The left side image illustrates a photonic pattern in the LC phase at 25 °C, while the right side image shows the same pattern in the isotropic phase at 120 °C. In the right section, the CLCPA material is wrapped around plastic tubing to demonstrate strain mapping [43]. (**b**) CLCPA temperature–color indication at 25 °C and 120 °C respectively [43]. (**c**) Reversible color change behavior of flower-patterned CLCPA under thermal stimulation [102]. (**d**) Structural color changes of bar-coated CLCPA during a warming–cooling cycle [10].

A demonstration highlighted the control over the color and shape reconfiguration of an AFT-capable CLCPA, showcasing spatiotemporal control over the material’s programmability and resulting in intricate patterns within the CLCPA [43]. Using a photomask with striped patterns of varying line spacings, the CLCPA was selectively patterned to create lines seemingly shifted towards blue. Upon heating, the patterned regions experienced a red shift; however, due to incomplete reversal, the lines appeared yellow/green in contrast to the red unpatterned regions (Figure 10a). Likewise, a chameleon skin display was created by patterning the CLCE with a uniquely shaped photomask to mimic the pattern of a chameleon’s skin. In the “relaxed” state, the pattern appeared blue and yellow, resembling the coloration of a chameleon’s skin. However, upon heating the material to its isotropic phase, it displayed green and red hues, akin to the coloration of a chameleon’s “excited” state (Figure 10b). Apart from being responsive to solvents, the reduced crosslink density imparted adequate flexibility to the coating, enabling it to undergo reversible color changes upon reaching the temperature for the cholesteric to isotropic phase transition [102]. This phenomenon was evidenced by the disappearance and subsequent reappearance of color upon exposure to elevated temperatures (Figure 10c).

To achieve significant shifts in the reflection band within a narrow temperature range, one strategy involves capitalizing on the transition from smectic A to cholesteric phases. This transition induces a remarkable and reversible unwinding of the helix, often termed the “pre-transitional effect” [103]. CLCPAs responsive to temperature changes based on this principle were synthesized [104,105,106,107]. A cholesteric liquid crystal exhibiting a temperature-dependent phase transition from smectic to cholesteric was synthesized [10]. Blade coating enabled the planar alignment of the cholesteric phase. When it is stabilized with a 3 wt.% crosslinked LC network, the photonic coating exhibits a color shift from red to blue when heated. The coating maintains high transparency, and the structural color alterations are fully reversible. At ambient temperature, the coating displays a subdued red hue due to decreased red reflection (partially in the infrared range) and slight scattering effects (Figure 10d). Upon heating, the coating undergoes a progressive thermochromic transition, shifting from 700 nm at room temperature (22 °C) to 480 nm (greenish-blue) at 53 °C, displaying a spectrum of colors in between.

**Figure 11:**
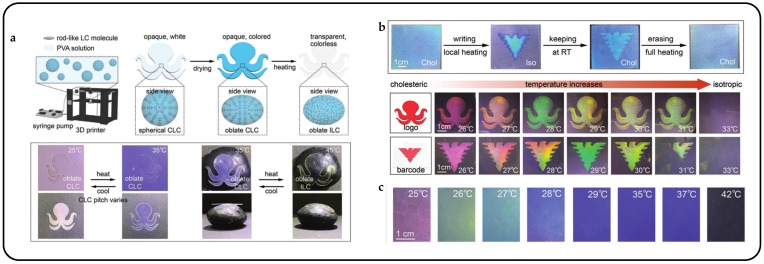
A 3D-printed biomimetic octopus exhibits synergetic responses in both color and shape. (**a**) Utilizing 3D printing technology, a CLC-droplet-dispersed polyvinyl alcohol (PVA) solution is printed into the shape of an octopus. Upon heating, the opaque and colored octopus becomes transparent and colorless [42]. (**b**) The process involves writing and erasing on oblate CLC-droplet-dispersed PVA films, enabling the creation of logos and anti-counterfeit barcodes [42]. (**c**) As the temperature increases, the structural color of CLC-dispersed droplets shifts to blue [42].

The coloration of 3D-printed biomimetic structures can be adjusted by modulating the helical pitch through variations in chiral dopant concentration or temperature. When heated to the isotropic phase, the oblate liquid crystal droplets, previously opaque and colored, transition to transparency and lose color in the biomimetic systems. At the same time, the isotropic liquid crystal droplets tend to assume a spherical shape, leading to volume contraction within the film plane and expansion perpendicular to it. This internal strain, combined with the gradient distribution of the oblate isotropic liquid crystal droplets, causes corresponding changes in shape (Figure 11a).

Inspired by the adaptive camouflage mechanisms observed in octopuses, CLC droplets were dispersed in a polymer solution and subsequently 3D-printed to create biomimetic structures [42]. During water evaporation, spherical CLC droplets changed into oblate shapes, selectively reflecting light through Bragg reflection in the central region. Similar to the pigment cells in octopuses, these oblate CLC droplets displayed structural coloration, modifiable by adjusting the chiral dopant concentration or temperature. Transitioning from oblate CLC droplets to oblate isotropic liquid crystal droplets causes changes in molecular alignment and droplet morphology, leading to simultaneous alterations in coloration and shape deformation (Figure 11b). Utilizing the unique characteristics of oblate CLC droplets, a variety of bioinspired stimuli-responsive systems have been developed, including intelligent logos and anti-counterfeit barcodes. Moreover, diverse biomimetic functionalities have been achieved, such as mimicking the camouflage of an octopus and the blooming of a flower. When oblate liquid crystal droplets were heated from the smectic phase, the gradual development of helical structures ensued, resulting in a reduction in helical pitch. This led to a blue shift in color, transitioning from red to blue, as depicted in Figure 11c.

**Figure 12:**
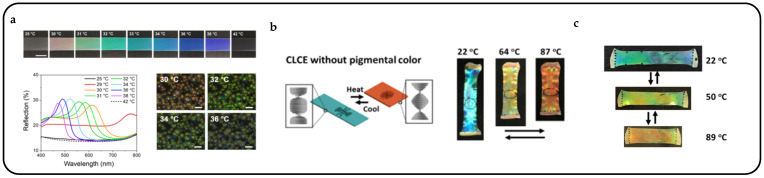
Thermal response CLC photonic actuator. (**a**) Illustrations of the temperature-responsive structural-colored CLCPA demonstrate color tuning within a small temperature interval (scale bar: 1 cm). Reflection spectra of the free-standing PDCLC/AgNW film upon heating, along with POM images (crossed polarizers) of the structural-colored CLCPA depicting the temperature-responsive color shift inside the spherical CLC microdroplets (scale bar: 50 μm) [109]. (**b**) Schematic illustration of reversible actuation and structural color change of the CLCE film, accompanied by photographs of a CLCE film without dye at different temperatures [110]. (**c**) Photographs of the CLCE film doped with IR 788 at different temperatures [110].

Low-molecular-weight CLCs can be encapsulated within a polymer matrix, leading to the formation of polymer-dispersed CLC systems [49]. These systems display reflective color shifts in response to temperature changes, which are attributed to the thermotropic nature of CLCs leading to variations in pitch length. Thermal response CLC droplets can be heated using a heater. The heater comprises a thin electrically conductive layer that efficiently generates rapid, controlled Joule heating by applying an electric current or potential to the surface area [108]. Arne A. F. Froyen et al. introduced multifunctional structural-colored electronic skins developed using scalable solution-processed methods, providing simultaneous monitoring of skin temperature and body motion through optical and electrical responses, respectively [109]. These multimodal photonic wearables integrated a temperature-responsive, freestanding photonic film with a thin, flexible conductive layer. The electrical output was established using silver nanowire (AgNW)-based ink, while photonic emulsions were employed to fabricate the freestanding structural-colored film (Figure 12a). Gradual heating of the photonic wearable induced a blue shift across the entire visible spectrum within a narrow temperature range, surpassing the smectic–cholesteric phase transition point of the thermosensitive CLC mixture. Notably, distinct colors were discernible for every 1 °C change in temperature, enabling these photonic wearables to detect subtle variations in body temperature through optical feedback.

Bending and color changes responsive to near-infrared light can be achieved through the utilization of a bilayer hydrogel comprising an inverse opal scaffold or a cellulose nanocrystal/polyurethane bilayer [111]. Pei Zhang et al. have successfully developed a pigmented structural color actuator utilizing CLCEs, demonstrating a lower actuation temperature and tunable responses to both temperature and near-infrared light for both actuation and coloration [110]. At 22 °C, the resulting CLCE film appeared green (Figure 12c), due to the combined effect of reflection by the cholesteric structure and light absorption by the embedded dye. To differentiate between the temperature-responsive reflection and absorption contributions, they fabricated a CLCEs film devoid of the near-infrared dye and a liquid crystal elastomer film lacking cholesteric order. At 22 °C, the resulting film exhibited a greenish-blue color due to reflection, appearing brighter compared to pigmented CLCEs. Upon heating to higher temperatures, the color shifts to red, a reversible process upon cooling (Figure 12b).

### 3.3. Electrical Response CLC Photonic Actuators

Electro-stimulated CLC actuators constitute a novel category of smart material devices capable of responding to external electric fields or electrical signal-driven devices, inducing shape or color changes. These actuators offer advantages such as rapid response, exact controllability, and low power consumption [112]. The electrical response CLC photonic actuator holds significant application prospects across fields such as information display and optical communication, thereby fostering new opportunities for the advancement of optoelectronic devices [113,114,115].

**Figure 13:**
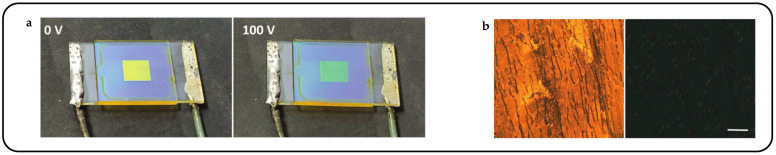
Images depict the behavior of liquid crystal cells under alternating current (AC) electric fields at different frequencies. (**a**) Photos illustrate the liquid crystal cell before and after the application of an AC electric field at 1 kHz frequency [13]. (**b**) Images demonstrate the electro-optic response variation of the double-layered cell under a high-frequency (1 kHz) AC field [13].

Electrically controlled liquid crystals serve as a transformative force in modern technology, reshaping various fields with their dynamic capabilities, particularly in displays and optical devices. In conventional liquid crystal displays (LCDs), electric fields are utilized to alter the orientation of liquid crystal molecules, thereby modulating the passage of light and generating high-resolution, high-contrast images [116]. Liquid crystal display devices utilizing electric field modulation have mature supporting systems, whereas CLC real-time structured color display devices do not rely on artificial light sources and can be employed in soft, low-power displays. Under the influence of an electric field, CLCs can not only undergo structural color changes but also adjust their transparency. In the fingerprint texture, the helical axis of the liquid crystals aligns parallel to the substrate, preventing selective reflection of incident light wavelengths and rendering the device transparent. In the homeotropic texture, liquid crystal molecules align in a directional order perpendicular to the substrate, facilitating transitions to other textures and rendering the CLC device transparent. Conversely, in the focal conic texture, the random distribution of CLC helical axes scatters incident light, resulting in non-transparency of the device. Bistable or multistable liquid crystal devices can be formed by utilizing electrically driven CLCs to transition between the aforementioned textures [50,117]. Their energy consumption is low, and they can be controlled within milliseconds. Alexey Bobrovsky et al. investigated the effects of electric fields and ultraviolet light on photochromic liquid crystal mixtures, analyzing their impact on the structure and performance of composite materials [13]. When subjected to an electric field, liquid crystal molecules aligned along the field direction, causing deformation of the helical structure [22,118]. Conversely, ultraviolet light induced the isomerization of azobenzene-containing molecules in a photochromic mixture (Figure 13a). These changes were reversible, with the initial state being restored upon deactivation of the electric field or exposure to visible light. The study also observed that the degree of polymer network swelling in a photochromic liquid crystal mixture is relatively low. Polarizing optical microscopy revealed the coexistence of planar oily streaks and marbled nematic textures, as shown in Figure 13b. After electric field-induced network swelling, the selective reflection band of the composite becomes switchable with high-frequency electric field application. Additionally, the electric field induced a shift of the center of the selective reflection peak to the shorter wavelength range, resulting in a change in color from red to green. This shift significantly affects only the long-wavelength shoulder of the selective reflection band, reducing the bandwidth approximately three-fold.

**Figure 14:**
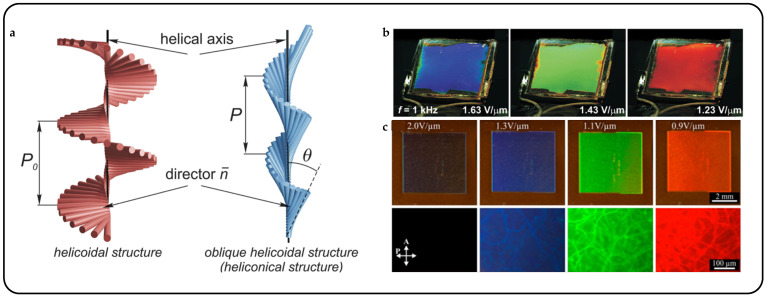
Oblique helicoidal cholesteric liquid crystals and electrically tunable structural colors. (**a**) Comparison diagram between CLC spiral nanostructures and oblique helicoidal CLC nanostructures [123]. (**b**) OHCLCs selectively shift their reflection wavelength towards a longer wavelength range under a reduced electric field [123]. (**c**) Electric field controlled selective reflection of light in DCM-doped CLC mixture, photos under ambient light conditions and corresponding polarized optical microscopy photos of DCM sample pool [124].

Synthesizing bent-shape flexible dimer molecules led to the experimental realization of a new cholesteric state characterized by an oblique helicoidal CLC structure, known as OHCLCs [119]. When a material is subjected to an electric or magnetic field, and its axis is parallel to the magnetic field [120], an OHCLC state is formed. The primary advantage of OHCLCs is that the pitch changes in response to the field, yet the single harmonic helical structure remains intact (Figure 14a). Without an electric field, the spiral directors rotate vertically with a specific pitch. When an electric field is applied, the spiral directors form an inclination angle. As the applied electric field decreases, both the pitch and the inclination angle of the helix increase [17]. In 1968, P.G. de Gennes [121] and R B. Meyer [122] predicted the unique response of CLCs to an external field, where the molecules rearrange from perpendicular to the helical axis *θ* = *π*/2 to a certain angle *θ* < *π*/2. A unique oblique helical CLC structure is formed by birefringent liquid crystals under the influence of an electric or magnetic field. This field modifies the pitch and cone angle of OHCLCs without altering the single harmonic modulation of the pointing vector. As a result, OHCLCs demonstrate remarkable effects, including electrically or magnetically tunable structural colors and lasers. The tunable range is extensive, allowing a single cell to shift its selective reflection wavelength from ultraviolet to visible light and further into the infrared spectrum.

Olena S. Iadlovska et al. demonstrated that the wavelength and bandwidth of selective light reflection on a tilted helical surface in OHCLCs can be controlled by surface alignment. When the alignment was vertical, the wavelength of Bragg reflection shifted to blue, and when it was planar, the wavelength shifted to red [125]. Mateusz Mrukiewicz et al. reported that by simultaneously irradiating the structure with ultraviolet light and applying an electric field, the structure color can be adjusted [126]. With newly designed rod-shaped, chiral, and curved azo photosensitive materials, doping cholesterols with oblique helical structures is characterized by a very low thermal back cis–trans isomerization rate. Under ultraviolet light, the cis–trans isomerization of photoactive compounds leads to a red shift in selective light reflection in cholesterol mixtures. Vitalli Chornous et al. induced helical distortion of the mixture of CB7CB/CB6OCB using the novel photosensitive chiral dopant ChD-3816 (an azo compound containing 4-hexyloxyphenyl and 2-isopropyl-5-methylcyclohexylbenzoate), and they added 5CB to reduce the phase transition temperature of the oblique helicoidal state of a twist–bend nematic doped with a chiral azo-compound [123]. Through experiments, they found that the CLC mixture can form OHCLCs under an AC electric field at temperatures higher than the phase transition. Molecules were observed to twist around the helical axis at a certain tilt angle, distinct from traditional CLCs. When subjected to an external electric field surpassing the threshold, the tilted helical structure transforms towards a vertically arranged helical structure. The dynamics of the transmission spectrum of OHCLCs as the applied electric field decreases are depicted in Figure 14b.

Jie Xiang et al. prepared a sample as a cell with an OHCLC plate with a thickness of 50 μm, which was confined between two glass plates with a transparent electrode coating [124]. Above a critical threshold, a strong electric field with an amplitude of ~2.0 V/µm unfolds the OHCLC into a uniform vertical texture, aligning its direction vector parallel to the electric field. This state appears dark between the cross polarizers. It does not have a photonic bandgap for laser emission. In a smaller field, the directional vector twists into an OHCLC structure, exhibiting selective reflection of light. This structure is electrically tunable across the near-UV to visible to near-IR range (Figure 14c).

**Figure 15:**
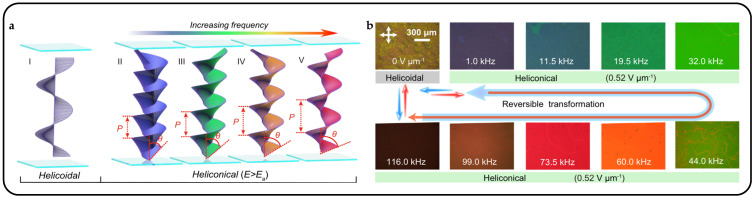
Schematic diagram of frequency-driven spiral soft structure at relatively low electric field intensity. (**a**) Schematic diagram of the influence model of frequency on nanostructures. (**I**) In the absence of an external electric field, LC molecules are perpendicular to the helical axis. (**II**) Increase the electric field signal with initial frequency and intensity, and the LC molecule revolves around the helical axis with an inclination angle of θ. The helix pitch length P and slope angle θ (**II**–**V**) increase with increasing frequency, resulting in tunable spectral range of wide reflection spectra. (**b**) At a fixed electric field intensity, as the frequency increases from 1.0 kHz to 116.0 kHz, the textures of helical and electro-induced helical microstructures exhibit different reflection colors [127].

In fact, the nanostructure tuning of OHCLCs is also related to the frequency of the applied electric field. Binghui Liu et al. established a frequency-driven helical soft structure, which is significantly different from common frequency-responsive soft materials [127]. Achieving reversible modulation of the photonic bandgap over a wide spectral range, they coupled frequency-dependent thermal effects, field-induced dielectric torque, and elastic equilibrium (Figure 15a). It is worth noting that continuously increasing the frequency while maintaining the electric field strength causes a significant redshift in the reflection band, exceeding 300 nm. This shift almost spans the entire visible light band, converging at the center wavelength of 760 nm, at a frequency of 116.0 kHz (Figure 15b).

**Figure 16:**
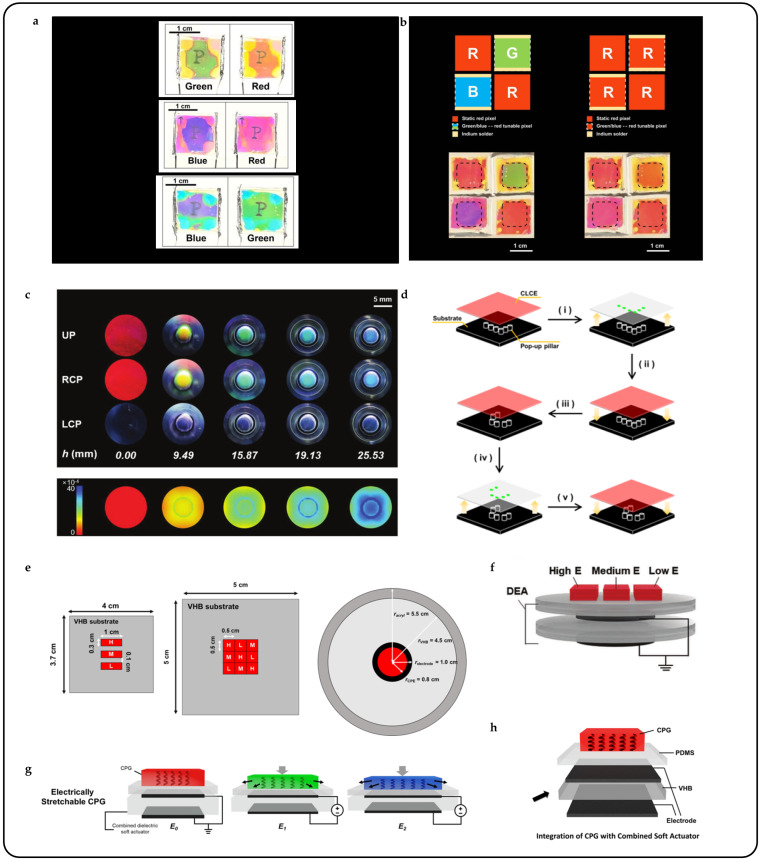
Electrical response CLC photonic actuator. (**a**) Actual operation images of the device [12]. (**b**) Pixel binning of the tunable color filter using heterogeneously structured CLCs [12]. (**c**,**d**) Electrically driven sensing/recognition/driven smart electronic skin system [137]. By (**i**) changing the height of the CLCE and (**ii**) lowering the substrate, (**iii**) changing the letters on the pop-up column of the substrate, (**iv**) when the changed substrate is raised again, and (**v**) reducing the height of the CLCE, different letters can be displayed. (**e**–**h**) CLCPA color changer driven on dielectric elastomers and chunked drive with multiple elastic moduli [56].

When current passes through the pre-designed resistive circuit, Joule heat is generated and radiated to the ambient environment, thereby driving the thermally responsive CLCPAs. Su Seok Choi et al. introduced an electrically tunable color filter utilizing a heterogeneous CLC structure. This innovation enabled the tuning of transmitted light across different colors (red, green, and blue) with voltages as low as 1.2 V [12]. Furthermore, analog pixel binning with a heterogeneous CLC color filter was investigated. Actual operational images of the fabricated voltage-induced CLC color filter devices, featuring the letter ‘P’ on the back sides of the devices, are depicted in Figure 16a. Each device could switch between red, green, and blue colors within the visible spectrum based on the applied voltage. In the actual operational images of the binning operation (Figure 16b), all color filters, initially displaying different colors, were adjusted to transmit red light based on changes in the applied voltage. Notably, devices placed at the top-right and bottom-left corners could simultaneously switch colors from green or blue to red based on the applied voltage.

An alternative method for electrically controlling the structural color change involves utilizing motors or signals to drive elastomers or actuators, inducing overall or regional deformation of a CLCPA. By precisely controlling the magnitude and direction of electric fields, researchers can fine-tune the helical structure of CLCs, thereby altering the wavelength of reflected light and producing vivid color changes [128]. Investigations were conducted on the optical characteristics of stretchable cholesteric liquid crystal elastomers (CLCEs) under out-of-plane stretching using a circular pop-up pillar, as illustrated in Figure 16c. The potential applications of stretchable CLCEs in conveying meaningful information were explored. This demonstrated successful control over the display of informative letters “LC” through the manipulation of equivalent out-of-plane stretching modes, as depicted in Figure 16d. This achievement was made possible by inducing a color change in the CLCEs from red to green during the out-of-plane stretching process.

A dielectric elastomer actuator (DEA) comprises an elastomer layer sandwiched between two flexible electrodes. The elastomer deforms in response to an applied voltage due to Maxwell stress induced by an electric field [129]. DEAs, as emerging next-generation flexible actuators, have demonstrated applications in micro-robotics [130,131], wearable haptic devices [132,133], and vibration isolation devices [134,135]. Controlling the vibration frequency and amplitude of a DEA enables directional control of the structural color pixel points of a CLCPA [56,57]. Figure 16h illustrates a multilayer drive structure for commercial elastomers/DEAs/CLCPAs, where the structure color was dynamically changed by applying AC power to the motor. Throughout a vibration cycle, the CLC displayed a reversible continuum of selective reflections shifting from red to blue (Figure 16g). Simple electrical tuning involved integrating CLCPAs with different elastic moduli onto the same commercially available elastomer substrate to create chunked display pixel blocks, as depicted in Figure 16e,f.

**Figure 17:**
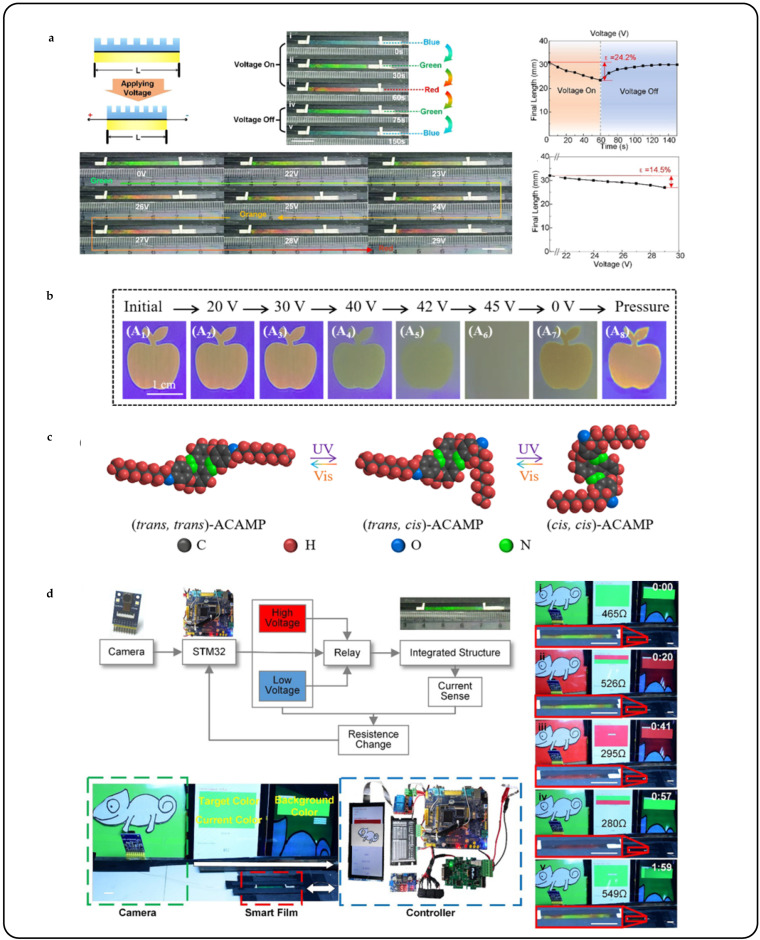
System-integrated bionic color change/deformation camouflage control. (**a**) Electro-joule thermally driven sensing/driven intelligent systems [138]. The structural color changes during the voltage on stage (**i**–**iii**) the voltage off stage (**iv**,**v**). (**b**) Images demonstrate the electro-optic response variation of the double-layered cell under a high-frequency (1 kHz) AC field [136]. (**c**) Isomerization of ACAMP [136]. (**d**) Electrically driven sensing/recognition/driven smart electronic skin system [138]. (**i**) is the initial state. (**ii**) and (**iii**) simulate color camouflage in a red environment. (**iv**) and (**v**) simulate color camouflage in a green environment.

Yuanyuan Shang et al. investigated the utilization of non-polymerized CLCs, blended with the main liquid crystal E7, axially chiral azobenzene-based molecules (ACAMP), and S-type chiral dopants (S5011), for generating photonic patterns with multimode memory effects [136]. The spatial photonic patterns were created by combining a PVA alignment layer with the photoisomerization of ACAMP. The patterns could be gradually erased by applying an electric field or raising the temperature to the isotropic state. Upon cooling or removing the electric field, the erased patterns reappeared with a vague hue. The electric memory mode was demonstrated through reversible electrical erasure, transitioning the molecular alignment from an orderly oriented planar state to a randomly aligned scattering focal conic state. Upon the removal of the electric field, the pattern retained a slight green hue due to the molecular transition from the randomly aligned scattering focal conic state to an imperfect planar texture (Figure 17b). Additionally, a pattern was generated by exposing the LC cell to 365 nm light, inducing reversible trans–cis photoisomerization of ACAMP, which altered the helical twisting power value and resulted in a red shift in the reflective stopband (Figure 17c). Reversal from cis–cis to trans–cis and trans–trans states could be achieved through visible light irradiation at different wavelengths, which produced distinct photostationary states (PSSs) and enabled wide photonic bandgap tuning of the CLCs.

Masters of disguise in nature rely not only on special skin structures that mimic anthropomorphic color changes but also on strong environmental perception, decision-making, and cognitive abilities. This coordination is reflected in artificial systems through sensor–processor–actuator integration. Weitian Zhang et al. aimed to develop a new intelligent material that mimics the color adaptation mechanism observed in chameleons [138]. The material comprised a photonic crystal film, liquid crystal elastomer, and carbon nanotube coatings, enabling various functionalities such as structural coloration, deformation, and sensing. When subjected to low voltage, the intelligent material underwent deformation, enabling the switching between red and blue hues (Figure 17a). The carbon nanotube coatings detected the deformation and provided feedback by changing resistance. These functionalities were leveraged to introduce a camera and controller system for constructing an active tunable structural color system. The system emulated the active tunable color mechanism observed in chameleons by utilizing an intelligent material integrated with structural coloration, sensing, and actuation functionalities. Furthermore, an image acquisition unit (to simulate eyes) and an information processor (to mimic brain function) were incorporated to enable autonomous color modulation in response to environmental stimuli (Figure 17d).

Throughout our exploration of electrically driven color-changing technologies, we have examined a diverse array of mechanisms and materials, each providing unique pathways for dynamic optical modulation and adaptive responses. Reflecting on the insights gained from our exploration, it becomes evident that electrically driven color-changing technologies hold immense promise for revolutionizing not only displays, robotics, and optics but also other fields. Such technologies offer unprecedented opportunities for creating interactive and responsive devices capable of adapting to evolving environmental conditions, user preferences, and functional requirements.

### 3.4. Light Response CLC Photonic Actuators

The increasing demand for multidimensional and dynamic control of light has driven the advancement of stimulus-responsive, reconfigurable, and programmable optical systems [139]. The core of optically controlled color change in CLCs resides in their intrinsic sensitivity to external light stimuli, especially in the visible and near-infrared spectral regions. The non-contact nature of light manipulation renders them suitable for designing optical devices that respond to visible/infrared light, exhibit tunable behavior, and modulate light [140].

**Figure 18:**
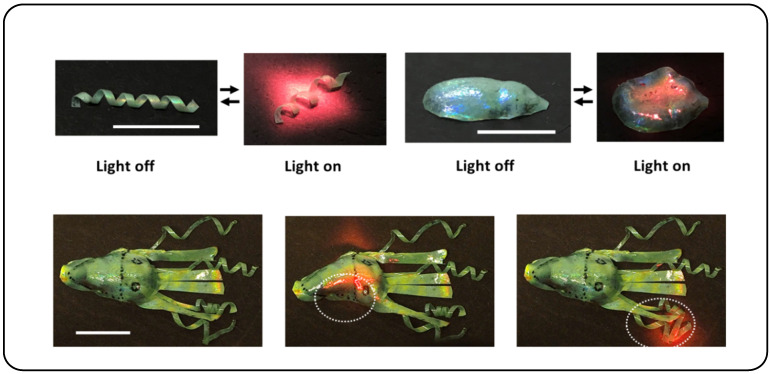
Cuttlefish irradiation under NIR produces morphological and color changes that mimic the behavior of organisms that tend to avoid hazards [110].

One approach for light-controlled actuation of CLCPAs is through a photothermal method [141,142,143]. Photothermal agents can selectively absorb specific wavelengths of light and efficiently convert them into internal energy. Typical photothermal agents comprise carbon nanotubes (CNTs) [144,145,146], metallic nanoparticles [147,148,149], plasmonic nanostructures [150,151], and reduced graphene oxide (RGO) [152,153,154]. The photothermal agent IR788 has been previously reported [155]. Pei Zhang et al. reported a CLC-based near-infrared light-driven 4D tinted structural color actuator that showed complex deformation [110]. Consequently, the CLC film displayed a spiral morphology with a green hue. Upon illumination with halogen light, the spiral structure transitioned to a red color and began to unwind, returning to its original state as the sample temperature reached 95 °C. By utilizing a 780 nm NIR light, localized unwinding of the spiral structure was achieved, attributed to a peak temperature of 72 °C. Moreover, a three-dimensional “cuttlefish” structure was fabricated by combining flat spiral and cone-shaped CLCE elements (Figure 18). Adhesive tape was used to attach the spiral arms of the CLCEs to the cone-shaped body. Upon activation of the halogen lamp, the spiral arms unwound, the straight arms contracted and turned red, and the body flattened and changed to a red color, reaching a temperature of 134 °C. Enhanced local control of the cuttlefish was demonstrated using a 780 nm NIR light. When the “head” of the cuttlefish was exposed to the NIR light, it promptly sensed the light and initiated contraction and localized color modification. Photocontrol based on the photothermal effect is fundamentally a form of thermal control, as described earlier. In this mechanism, the driving force behind the structural color change relies on both the conversion efficiency of the photothermal agent and the response time of the actuator.

**Figure 19:**
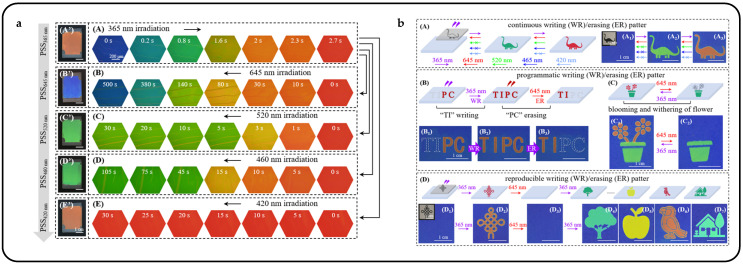
Light response CLC photonic actuator. (**a**) Under POM, the reflection color changes upon irradiation with 365 nm light at various durations, followed by the reverse process upon irradiation with visible light at different wavelengths: 645 nm, 520 nm, 460 nm, and 420 nm, as depicted in (**A**–**E**). Corresponding real LC cell images are provided in (**A’**–**E’**) to illustrate the red, blue, green, green, and red photostationary colors of the light-driven CLCs at PSS365 nm, PSS645 nm, PSS520 nm, PSS460 nm, and PSS420 nm, respectively [136]. (**b**) Schematics and photographs of the patterns demonstrate precise control of reflection color programmed by 365 nm/645 nm light irradiation [136].

Certain photochemically light-responsive substances can undergo photoisomerization upon exposure to specific wavelengths of light. This remarkable reaction enables CLCs to become photo-tunable. Several photoisomerized substances, including spiropyran [156,157,158,159], diarylethenes [160,161,162], and azobenzene [163,164,165,166,167,168], have been reported to undergo reversible changes in geometry. Table 2 describes a selection of commonly used photoisomer substances. Spiropyrans can be converted from closed to open rings under UV irradiation conditions, and this isomerization is stable and can be reversibly transformed by visible over-irradiation or heating [169]. Diarylethenes and their derivatives can also accomplish open- and closed-loop transitions under UV light irradiation [161]. The presence of cis–trans photocontrol molecular heterostructures in azobenzene [170] and its derivatives makes them ideal media for light-driven soft actuators as well as chiral optical switches [171,172]. Under UV irradiation, azobenzene can be converted from the trans isomer to the cis isomer and can be transistorized by thermal reversal or visible light irradiation. Yuanyuan Shang et al. realized versatile photonic structured color patterns using axially chiral azobenzene molecules mixed with CLCs [136]. The irradiation of light initiated the photoisomerization of azobenzene, inducing changes in HTP and providing the opportunity to generate colorful patterns through controlled adjustment of CLCs’ structural color. The reflection color of the samples continuously shifted towards the desired hue upon irradiation with 365 nm light for varying durations, resulting in a transition from blue to red in the exposed regions, while the unexposed areas retained their blue color (Figure 19a). Upon irradiation with 365 nm light, the reflection color of the sample transitioned from blue to green within 0.8 s, and further to red within 2.7 s. Subsequently, the red sample underwent a complete and reversible shift to blue within 500 s upon irradiation with visible light at 645 nm. Notably, irradiation with 520 nm light induced a blue-to-green color shift within 5 s, while irradiation with 460 nm light prompted a similar shift within 45 s. However, minimal change in reflection color was observed under 420 nm irradiation. Figure 19b shows the structural color reflections of patterns as well as text under different specific wavelengths of light stimulation. Azobenzene provides the potential for pattern memorization in its response to light and heat, while the photoisomerization effect enables patterns to be erased and rewritten repeatedly, presenting novel possibilities for the utilization of photonic materials.

**Figure 20:**
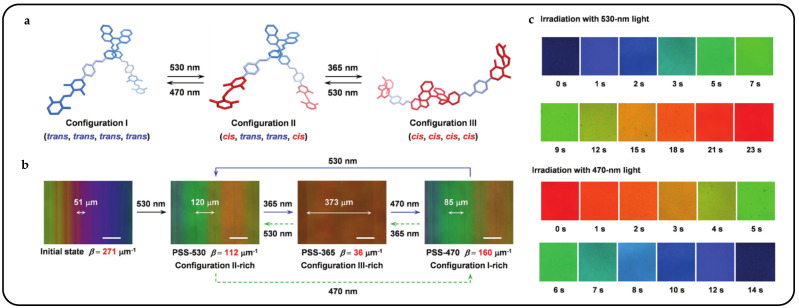
Under polarizing optical microscopy. (**a**) Schematic illustration showing the optimized molecular structures of three configurations of the chiral switch [177]. (**b**) Optical images showing the disclination lines of 0.19 mol% chiral switch in LC host E7 at different states [177]. (**c**) Reflection color images of 2.0 mol% chiral switch in LC host E7 in a 5 μm thick antiparallel aligned cell [177].

Designs utilizing azobenzene and its derivatives can be combined to achieve sophisticated light control designs. Lang Qin et al. introduced intricate patterns comprising three primary red, green, and blue (RGB) colors against a black background, achieved through the segmented reflection tuning of CLCs using a newly developed photoresponsive tristable chiral switch [177]. By integrating two distinct photoswitches into a single chiral structure, they established a novel chiral switch. Notably, the significant variation in *HTP* across the three configurations of the chiral switch enables the CLCs to exhibit two continuous and adjacent tuning periods of reflection (Figure 20a). These periods could be independently controlled by light of different wavelengths (Figure 20b). While maintaining the RGB reflection colors within the visible spectrum, the reflection was extended into the near-infrared region to produce a black background (Figure 20c). This unprecedented piece-wise reflection tuning of self-organized helical superstructures in the CLC, driven by the twistable chiral switch, introduced a novel concept for RGB and black reflective displays.

Light control serves as a cornerstone across various domains, encompassing scientific inquiry, engineering endeavors, and everyday technological applications. Within this expansive landscape, the capability to manipulate light (intensity, wavelength, polarization, etc.) opens the door to a myriad of functionalities crucial for advancing research and facilitating innovative technologies. This precise manipulation facilitates the tailored engineering of CLC structures, enabling dynamic color tuning and adaptive optical responses customized to specific application requirements.

### 3.5. Humidity/Magnetic/Pneumatic Response CLC Photonic Actuators

Humidity sensors play a vital role in measuring the relative humidity of air, offering valuable insights into moisture content under specific temperature and pressure conditions [178,179]. Given the rising demand for gas monitoring across industrial sectors and daily activities, advancements in humidity sensor technology are crucial [180]. Traditional inorganic semiconductor-based sensors face limitations such as slow response times, limited selectivity, and high operating temperatures [181,182]. In response to these challenges, CLCPA humidity sensors have emerged [183,184,185,186], utilizing intricate periodic structures. These optical sensors have become popular due to their high sensitivity, low power consumption, and ease of manufacturing. Wenzhu Cao et al. devised a straightforward, efficient, and battery-free visual humidity sensor by blending CLCs with acrylic acid and subsequently curing them to create an interpenetrating polymer network (IPN) film, capable of changing color with relative humidity [187]. Polyacrylic acid (PAA) is renowned for its humidity sensitivity, often integrated into liquid crystal film networks, thereby imparting the resulting interpenetrating polymer network (IPN) with the remarkable capability to undergo optical transformation in response to humidity changes. The fundamental mechanism of this IPN involves the absorption of moisture by the PAA, leading to its swelling, and the subsequent exertion of a force that triggers a corresponding swelling within the LC film [188].

**Figure 21:**
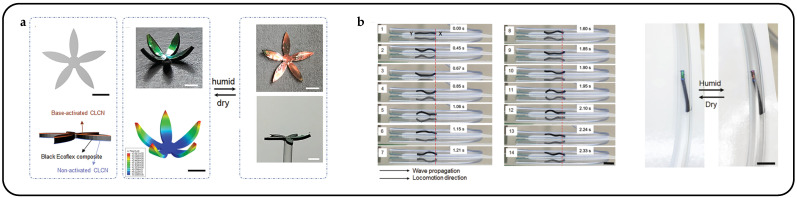
The coordination of an artificial flower in the humid air environment. (**a**) The shape curling and color displacement of artificial flowers in dry/humid environments (scale: 3 mm) [39]. (**b**) With the help of fluctuations and friction in the rotating magnetic field, the actuator facilitates forward translational motion inside the tube and the color change caused by humidity in a robot used to sense humidity in a restricted tube (scale: 10 mm) [39].

Interpenetrating polymer networks (IPNs) and semi-interpenetrating polymer networks (SIPNs), comprising two distinct yet intertwined polymer networks, thereby amalgamating the advantages of both components, have been regarded as strategies for the preparation of novel CLCPAs [189,190,191]. Wei Feng et al. introduced an innovative approach for fabricating a dual-responsive elastic cholesteric polymer material by establishing a semi-interpenetrating network (SIPN) comprising a cholesteric main-chain polymer and a hygroscopic poly(ampholyte) [39]. This material demonstrated a redshift in reflected color upon water swelling and a blueshift under mechanical strain. Leveraging the mechanochromic behavior of CLCEs and the water-absorbing/desorbing capabilities of the poly(ampholyte) network, the film underwent color changes not only during stretching and relaxation but also due to volume changes induced by water swelling. Out-of-plane actuation of the colorful actuators and decoupling between deformation mode and shape/pattern were demonstrated using a five-petal artificial flower with one side treated at the base (Figure 21a). In the dry state after the base treatment, the artificial flower curled due to asymmetric base processing and shrinkage. Upon exposure to moisture, the hygroscopic LC network layer underwent swelling and expansion, causing the curled flower to “bloom” and flatten, resulting in a color change from green to red. Integrated with magnetic control, the mobile sensor could navigate through open and confined spaces, aided by friction, to detect local relative humidity (Figure 21b).

**Figure 22:**
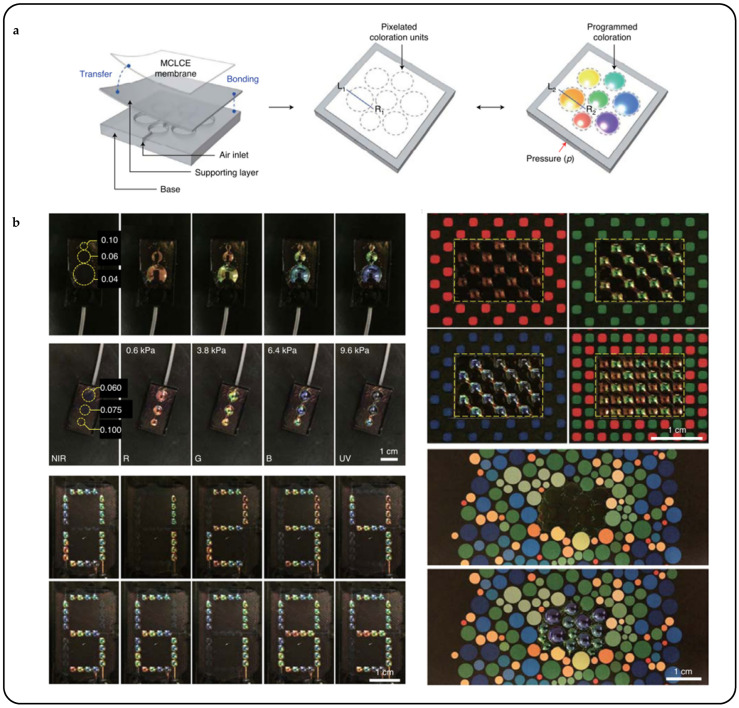
Pixelated artifacts of micro airway-controlled CLCPA. (**a**) Multilayer micro-airway model to escape gas-pressure-controlled CLC membrane deformation [192]. (**b**) Example of pixelated camouflage based on micro-airway control [192].

The utilization of gas-driven CLCPAs for structural color camouflage represents an innovative approach. A novel approach was introduced to modify the color response of thin membranes consisting of CLCEs through pneumatic inflation [192]. Strategically designing the size and layout of enclosed air channels within these membranes enables color modulation spanning from near-infrared to ultraviolet wavelengths, with minimal biaxial transverse strain not exceeding 20%. Each channel serves as a controllable “pixel”, enabling precise color adaptation to surrounding environments, regardless of whether the patterns are periodic or irregular (Figure 22). The principle behind inflation-induced pixelated camouflage relies on the ability of CLCPAs to bend or swell in response to external stimuli, such as changes in pressure or mechanical forces. Introducing microfluidic channels or compartments filled with a CLCPA-containing fluid allows researchers to manipulate the inflation and deflation of individual pixels within a camouflage system, enabling precise control over color and pattern transformations.

This section is summarized in Table 3. Discoloration behavior in humidity-responsive CLCPAs can be achieved through bending or swelling caused by uneven humidity or moisture absorption. By capitalizing on the unique properties of CLCPAs, researchers can continue to innovate and develop new technologies that leverage humidity-responsive materials [193,194] for a variety of practical applications. Besides their humidity-responsive behavior, cholesteric liquid crystal photonic actuators (CLCPAs) can also demonstrate responsiveness to magnetic fields, presenting intriguing possibilities for dynamic modulation and control [195]. Introducing magnetic substances into the CLC system enables the engineering of CLCPAs to respond to specific magnetic field environments, thereby broadening their utility in various applications. The magnetic field-responsive behavior of CLCPAs shows promise for various practical applications, such as magnetic field sensing, actuation, and manipulation. Leveraging the responsiveness of CLCPAs to magnetic fields, researchers can develop innovative magnetic field sensors and actuators capable of detecting and responding to changes in magnetic field intensity and orientation. Researchers are exploring novel strategies to achieve pixelated camouflage through controlled inflation and deflation processes, harnessing the unique properties of CLCPAs.

## 4. Advanced Optical Applications

Having elucidated the surprising structural color change ability of CLCs as well as multiple conditional actuator deformations and their fabrication techniques, our attention shifts to an in-depth study of their advanced optical applications. CLCPAs’ unique color change has great potential for application development in colorimetric sensing, smart wearable fabrics and devices, advanced color filters, novel 3D printing, optical cloaking, and camouflage.

### 4.1. Colorimetric Sensing

In the realm of sensing technologies, the advent of visually interpretable colorimetric sensing has marked a significant paradigm shift, offering a compelling alternative to traditional sensor approaches. Naked-eye recognizable colorimetric sensing uses the human eye’s ability to perceive color to enable the perception or amplification of other physical quantities. In such systems, no microscope or signal processing equipment is required, but rather the structural color changes produced by the sample are analyzed by direct observation with the naked eye. Compared to complex instruments, colorimetric sensors that are recognizable to the naked eye are simple to operate and suitable for non-precise daily life scenarios and do not require specialized training to use. The real-time feedback feature of a CLCPA makes it important in time-sensitive scenarios that require fast feedback and real-time monitoring.

**Figure 23:**
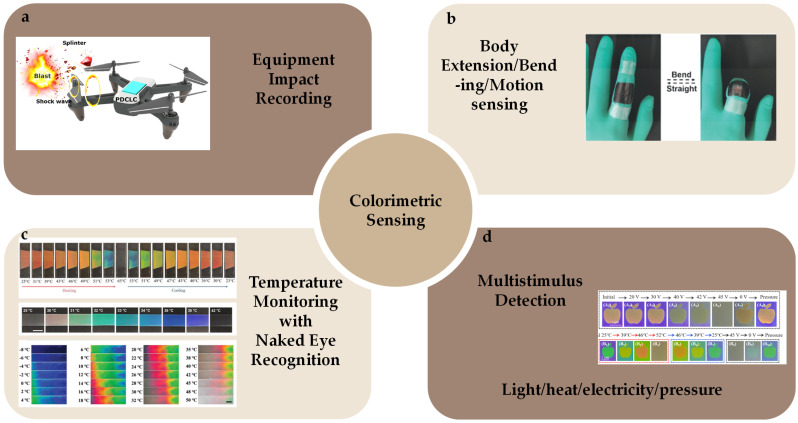
Colorimetric sensing in equipment impact recording and body extension/bending/motion sensing as well as single- and multiple-stimulus detection. (**a**) Colorimetric sensing for mechanical recording: the impact of explosives and shock waves on UAVs [44]. (**b**) The bending of finger joints causes the colorimetric sensor to change the structural color, thereby determining joint flexibility [80]. (**c**) Temperature induced significant structural color changes in CLCPA [10,99,109]. (**d**) CLCPA responds to color changes in multiple physical quantities such as light, heat, electricity, and pressure [136].

Structural color changes caused by changes in the helical nanostructures of CLCs subjected to external mechanical forces are the most direct form of sensing stress–strain. In the military and security field, CLCPAs can be used to monitor and assess the destructive force of explosives strikes on military equipment. Applying CLC photonic coatings to the surface of military equipment and facilities can allow the forces on the equipment to be checked in real time, especially for destructive tests with limited repeatability or high cost, such as directional blasting, to improve the efficiency of research and development of military equipment and facilities. Zhao Xu et al. proposed that shockwave and debris impacts on unmanned aerial vehicles (UAVs) could be recorded by applying a CLC photonic polymer coating [44] (Figure 23a). Collecting information about blast-exposed impacts made point-to-point protection of UAVs possible. CLCPAs’ short-term storage of mechanical information and unique polarized spinning properties could be applied to optical information storage [45,196] as well as information encryption [197]. Since a CLCPA, which changes color due to stress–strain stimulation, could be subjected to various forms of deformation such as stretching, bending, and torsion, it could be used for the detection of body and joint movements [80] (Figure 23b). CLCPAs’ vibrant structural colors could be used for a wide range of temperature detection up to 50 °C [10,99,109] (Figure 23c). Visible shape/color changes could be observed during temperature changes. These temperature measurements were sensitive in real time, but were non-precise and could be made into alarm tags to detect reaction temperatures in the production process. These close-to-human-temperature applications were also promising for medical and health testing, biomedical instrumentation, and other fields. In addition to a single physical quantity such as temperature, simultaneous colorimetric detection of multiple stimuli could be achieved by doping the CLC system with azobenzene and other stimulus-corresponding agents and combining it with a multifunctional actuator [136] (Figure 23d). This feature empowers CLCPAs to write soft sensors for complex and specialized environments that are expected to be lightweight and customizable.

### 4.2. Smart Wearable Fabrics and Devices

**Figure 24:**
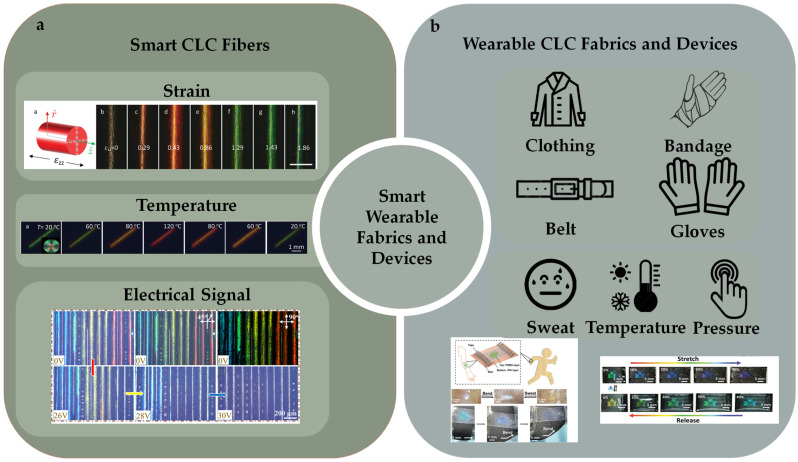
Smart wearable fabrics and devices. (**a**) Structural color response of smart CLC fibers to strain, temperature, and electrical signals [205,206,207]. (**b**) Wearable CLC fabrics and devices: clothing, bandages, belts, and gloves can detect sweat, temperature, and pressure [208].

The design and development of wearable sensor systems based on flexible fibers for measuring physical and chemical signals from the human body as well as from the environment offers applications for disease diagnosis, treatment, health monitoring, and safety [198,199,200]. Wearable smart fibers and devices have been used in a variety of flexible wearable platforms such as clothing, bandages, belts, and gloves (Figure 24b) to monitor important body characteristics such as temperature, perspiration, and pressure [190,201,202,203,204]. Flexible CLCPAs have multiple response modes to motion, temperature, and humidity, with small size and portability, providing opportunities for safety monitoring, disease diagnosis, and physical condition monitoring. Md Mostafa et al. developed cholesterol liquid crystals dispersed in commercial water-based latex varnish coated on fibers and fabrics [205] (Figure 24a). The color of CLC latex coatings varies with temperature and could be applied to wearable temperature monitoring. This ingenious design enabled rapid and uniform color changes, maintaining stability over extended periods. Mingfei Sheng et al. presented an electrochromic liquid crystal cladding (ECLCC) optical fiber, which was the incorporation of various electrochromic materials, notably cholesteric liquid crystals, within a unique coaxial double-reverse electrode sandwich structure [206]. Through innovative weaving techniques, long-sized ECLCC fibers were seamlessly integrated into textiles, opening up a plethora of possibilities for wearable flexible displays. Celina Jones et al. reported on a solution for inkjet printing of CLC onto hydrophobically pretreated textiles as an alternative to existing chemical dyeing techniques [207]. The resulting colored films showed a greater degree of color than those on untreated textiles. In addition, the inkjet printing process allows the printing of a predefined dose of CLCs in a precise location, and it is possible to digitally print designs containing a variety of high-precision color patterns using a solution that changes the structural color simply by varying the amount of chiral dopants. Smart fabrics composed of smart soft fibers based on CLCs can intuitively display vibrant patterns and sense temperature through changes in structural color, making them an eye-catching class of materials.

In addition to their role in colorimetric sensing, CLCPAs are ushering in a new era of innovation in flexible wearable devices. Pressure sensors based on sensitive force deformation represent a promising avenue for leveraging CLCPA technology in wearable devices [44,209]. These sensors have been explored for various applications, including exercise monitoring, healthcare diagnostics, and positional detection [210,211]. By incorporating CLCPA elements into pressure-sensitive materials, such as thin films or gels, researchers can develop wearable sensors capable of accurately detecting and quantifying external forces.

In the realm of human pressure sensing, the configuration and placement of sensors play a crucial role in determining their efficacy. For instance, gloves equipped with CLCPA sensors can be utilized for finger curvature detection in postoperative rehabilitation settings, providing valuable feedback on range of motion and joint flexibility. Similarly, CLCPA-integrated shoe soles can measure the distribution and magnitude of forces during walking, enabling the diagnosis of abnormal gait patterns indicative of underlying health conditions. The versatility of CLCPA technology lends itself to the development of highly customizable and adaptable wearable devices tailored to specific applications and user needs. By combining colorimetric sensing capabilities with flexible and wearable form factors, CLCPA-enabled devices have the potential to revolutionize healthcare monitoring, athletic performance tracking, and rehabilitation therapy. In addition, CLCPAs can be used in smart wearable fabrics and devices for temperature, sweat, motion, and gas detection.

It should be noted that most applications of wearable flexible CLCPAs have been used to monitor user body parameters, but functions such as precise measurement and early warning of these parameters remain under-explored. Indeed, we predict promising developments in flexible sensor platforms for CLCPA-based sensing as well as response actuation system integration.

### 4.3. Advanced Color Filter

**Figure 25:**
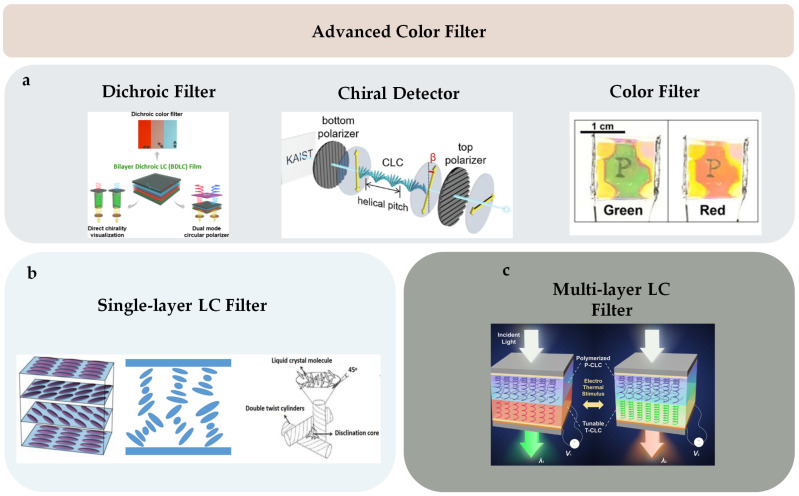
Advanced color filter. (**a**) CLCs are used in dichroic filters, bimodal circular polarizers, and chiral detectors [213]. (**b**) The basic structure of single-layer LC filters, which are simpler to fabricate than multilayer LC filters [215]. (**c**) The structure of multilayer LC optical color filters [12].

Novel optical filters can extract useful information from massive optical signals on demand and are widely used in spectroscopy, complex imaging, and optical communications. Conventional color filters use the absorbance properties of pigments to selectively transmit incident light, which affects their optical quality [212]. CLC templates have emerged as promising candidates for addressing these needs, offering a host of advantages over traditional filtering devices. CLC templates exhibit enhanced reflectivity compared to their counterparts, making them well-suited for applications requiring high optical efficiency. The unique photonic properties of CLC structures enable the generation of multiple reflection bands, allowing for precise control over the transmission spectrum. This flexibility enables the customization of filters to specific wavelength ranges, accommodating the diverse needs of modern optical communication systems. Additionally, CLC templates offer the flexibility to adjust reflection bands by refilling with different materials. This versatility allows for dynamic tuning of filter characteristics to adapt to changing requirements or optimize performance for specific applications. Geonhyeong Park et al. reported a CLC prepared using the uniaxial alignment method and a photopolymerization process that can be used in dichroic filters, bimodal circular polarizers, and chiral detectors [213] (Figure 25a). Wongi Park et al. proposed an optical rotation-based color tuning method, a cost-effective and non-destructive approach that controlled CLCs as chiral photonic crystals and two polarizers to provide a wide range of structural color tunability [214]. Tunable color filters are shown to attenuate accidental light sources and provide better visibility. Su Seok Choi et al. presented an electrically tunable CLC color filter using a heterogeneous helical nanostructure in which the transmitted light can be tuned between different colors with a voltage not exceeding 1.2 V [12] (Figure 25c). In order to obtain the electrical controllability of the transmitted color, the chiral liquid crystal was electrothermally tuned.

Single-layer LC filters are simpler to fabricate than multilayer LC filters. Zhikang Zhu and colleagues have demonstrated the fabrication of single-layer LC filters capable of generating multiple reflection peaks [215] (Figure 25b). This innovative approach involved filling CLCs with chiral spacings differing from those of the target template into a blue phase liquid crystal (BPLC) template. By leveraging this method, researchers could achieve a diverse range of reflection peaks within a single filter structure. The use of multiple templates and a refilling process offered the potential for further increasing the number of reflection peaks, enhancing the versatility and functionality of the filters. The sequential filling of CLCs with specifically designed chiral pitches into CLC templates enabled the fabrication of bandwidth-scalable single-layer CLC filters. This approach allowed for the precise control of filter characteristics, including bandwidth and spectral response, facilitating the optimization of filter performance for various optical communication applications.

CLC color filters have the advantages of low power consumption, low cost, various driving methods, and a simple fabrication process and have a broad application prospect. Due to the periodic helical structure, CLCs exhibit selective reflection of incident light. A CLC color filter has a sub-micron pitch, which makes it a great potential for application in mid-wave infrared filters. A CLC color filter is optically isotropic, does not require a polarizer or a dielectric layer, and has the advantage of sub-millisecond response time.

### 4.4. Novel 3D Printing

**Figure 26:**
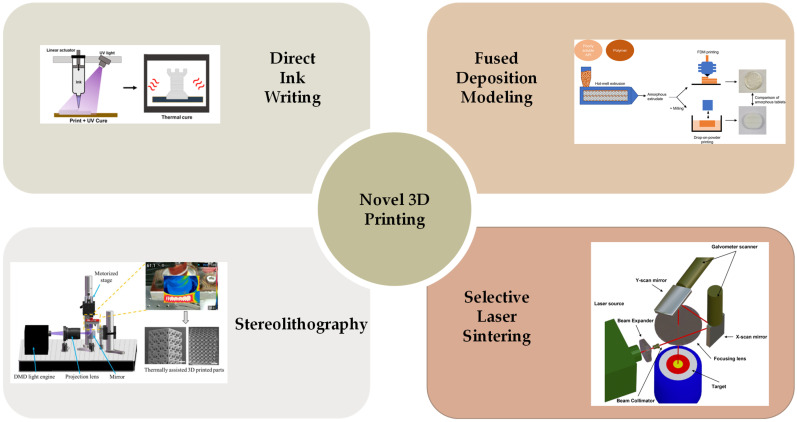
Typical 3D printing technology: direct ink writing (DIW), fused deposition modeling (FDM), stereo-lithography (SLA), and selective laser sintering (SLS) [216,217,218,219,220,221,222,223,224,225,226].

Three-dimensional printing technology, a bottom-up manufacturing process that builds various structures and complex geometries by printing layer by layer, has been widely used to realize patterned CLCPAs. With the help of 3D printing technology, CLCPAs can be constructed not only in pre-designed 3D models but also with controlled stimulus response. As a result, actuated shape deformations obtained by 2D–3D or 3D–4D transformations of external stimuli can also be precisely controlled, providing an easy manufacturing tool for engineers in different fields. Three-dimensional printing technologies in use today include direct ink writing (DIW) [216,217,218,219], fused deposition modeling (FDM) [220,221,222], stereolithography (SLA) [223,224], and selective laser sintering (SLS) [225,226] (Figure 26). Printing inks for 3D printing can be prepared by reacting CLC oligomers with chain extenders and crosslinking agents. The printing ink behaves as an adjustable viscous liquid and is stacked in layers on a carrier table after being ejected through a nozzle. Using the direct ink writing (DIW) technique, the print size can reach a centimeter scale, and the actuation properties of CLCPAs can be well controlled. Ran Bi et al. proposed a simple method to fabricate structural color patterns with wider resolution and enhanced scalability by solvent-cast direct ink writing (SC-DIW) technique under mild conditions [227]. Shear flow and anisotropic desolvation were utilized to form regular nanospiral structures. Printable inks were prepared by adding a volatile solvent to the synthesized CLC oligomers to form a viscous solution. Jihye Choi et al. found that the helix axis was skewed in the printing direction due to a combination of shear-induced alignment caused during extrusion and elongation forces generated during deposition onto the substrate [228]. This unusual spiral axis distortion caused blue and red shifts in the reflected colors, depending on the viewing direction relative to the print axis. Jeroen A. H. P. Sol et al. prepared reactive CLC oligomer inks that generated complex, spatially defined photonic patterns that produced intuitive visual effects [229].

To achieve untethered and fully controllable shape-shifting, researchers have integrated CLC ink with various additives, enhancing their responsiveness to external stimuli. Azobenzene CLCPAs are synthesized by introducing supramolecular interactions, reversible covalent crosslinking, and azobenzene into the same system [230]. Furthermore, there is a growing demand for real-time feedback mechanisms to monitor shape deformation and other properties during printing processes. Materials for 3D printing by doping CNT [231] and carbon black [232] have also been reported to have a controlled and sensitive response to embedded stimuli. Recently, liquid metals have been widely doped with CLC inks because of their high electrical and thermal conductivity [233,234]. Overall, 3D printing of CLC structures represents a convergence of materials science, engineering, and design, offering new possibilities for innovation and creativity in a wide range of applications. With continued research and development, these technologies hold the potential to transform the way we perceive and interact with color, enabling the production of dynamic and adaptive systems that respond to environmental stimuli and user interactions.

**Figure 27:**
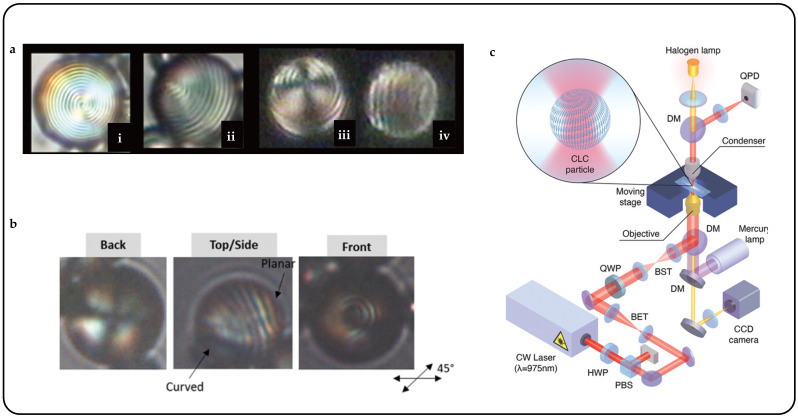
CLC micrometer-sized droplets and actuators. (**a**) Optical microscopy images of chiral microspheres from precursor long-pitch LC droplets (obtained in pure distilled water and mixtures of water and surfactants) [236]. (**i**) Images of microspheres with 1 μm spacing obtained in pure distilled water using lotion. (**ii**–**iv**) Image of microspheres obtained in a mixture of water and surfactants. (**iv**) Microspheres are organized into parallel layers of cholesterol. (**b**) POM images of large-pitch particles [237]. (**c**) Schematic illustration of the developed optical setup (see the main text and methods for the details). Inset image: schematic representation of an optically trapped CLC microparticle [239].

Liquid crystal polymer particles can be prepared using classical polymerization methods such as suspension polymerization, miniemulsion polymerization, dispersion polymerization, and precipitation polymerization [235]. Liquid crystal polymer particles, prepared using classical polymerization techniques, may find application in various fields including microactuators, structurally colored objects, and 3D printing. Gabriella Cipparrone et al. proposed new solid chiral microparticles, which are produced through a very simple soft matter self-assembly process [236] (Figure 27a). By varying the type of dopants in the precursor LC emulsion, it is possible to control the internal helical geometry, enabling the creation of solid microspheres with helical structures in radial, conical, or equatorial configurations. These structures can exhibit either global optical isotropy or anisotropy. Alberto Belmonte et al. fabricated micron-sized polymer particles with reversible shape and optical changes that respond to both light exposure and temperature variations [237] (Figure 27b). They selected a CLC mixture containing a small amount of crosslinker and synthesized the particles using suspension polymerization. During the emulsification process, they added an ionic surfactant to the water to impose homeotropic anchoring, resulting in a globally anisotropic arrangement of the helical structures. The rotational dynamics of solid chiral birefringent microparticles induced in optical tweezers have been studied [238]. Yera Ye. Ussembayev et al. experimentally and theoretically investigated the rotation of these polymer particles in optical traps [239] (Figure 27c). Due to their chirality, the polymer particles respond to the handedness of circularly polarized trapping laser beams, exhibiting unidirectional or bidirectional rotation depending on their alignment within the optical tweezers. The resulting optical torque induces rotation at rates of several hertz. Small structural changes induced by UV light absorption enable control over the angular velocity.

### 4.5. Optical Cloaking and Camouflage

In nature, the structural colors of living organisms usually have a bright and distinctive appearance that contrasts with the common coloring of dull conventional colors. The microstructure of organisms that have evolved over millions of years, filtered by nature’s law of survival of the fittest, has been repeatedly tested and optimized in the process to obtain the most suitable color mimicry. Animals can use shading or color information to perceive depth and 3D shape [240,241]. Cuttlefish [242], praying mantises [243], and honeybees [244] can use stereo vision to optimize shading camouflage to attack prey or avoid damage [245]. In the optical camouflage being applied today, patterned or pixelated blocks of dark color are used to simulate shadows because the camouflage coating is non-dimensional. Research has shown that spatial color mixing is one of the main features of digital camouflage and the key to improving the deception effect of the human eye [246]. Unlike the pseudo-3D visual deception in the two-dimensional plane, CLCPAs can not only achieve the visual–spatial illusion of color mixing but also simulate the real 3D shape through the pre-designed movements of the actuator, such as curling and bumping [247]. Optical cloaking and camouflage, which mimic the CLCPA structural colors of natural organisms, reveal new applications in military, communication, and remote sensing. By leveraging CLC-based camouflage materials, objects can seamlessly blend into their surroundings, evading detection by adversaries or surveillance systems. CLC-based camouflage materials have the potential to enhance urban camouflage, enabling urban infrastructure or equipment to seamlessly integrate into the surrounding urban environment.

**Figure 28:**
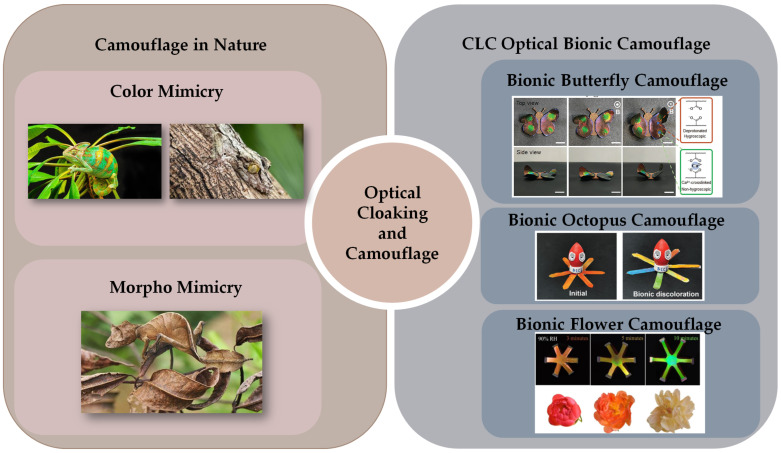
Camouflage in nature and CLCPA for optical cloaking and camouflage (butterfly [39], octopus [248], flower [84]).

One of the key features of structural color camouflage is its ability to dynamically adapt to changing environmental conditions and background textures. By incorporating responsive materials into camouflage structures, researchers can create adaptive systems capable of altering their appearance in real time. Hyeonseok Kim et al. integrated a thermochromic CLC layer with a patterned silver nanowire heater into a multilayer structure, combined with an active control system and sensing unit, to create a complete biomimetic chameleon system. This model has successfully imitated the background color of the environment and can quickly achieve dynamic matching [100]. Dongpeng Sun et al. fabricated a bionic flower with a metal–organic skeleton (MOF) and a CLCE multilayer structure to realize a blossoming action and structural color change [84]. Chang Sun et al. demonstrated a bionic model of cephalopods, showing color changes in CLC tentacles with length and environmental camouflage [248] (Figure 28). Structural color camouflage represents a cutting-edge approach to concealment and optical manipulation, offering unprecedented levels of adaptability, functionality, and security. By harnessing the principles of structural coloration, researchers are poised to unlock new frontiers in camouflage technology and optical engineering, paving the way for a future where invisibility and deception are achieved through the power of light and materials.

## 5. Conclusions and Outlook

This paper provides an overview of the different structures of CLCPAs and their structural color response to different stimuli, including mechanical force, heat, electricity, light, humidity, magnetism, and gas pressure. This paper also provides an overview of the emerging optical applications of CLCPAs. With their vibrant structured colors and controlled optical properties, CLCPAs are a landmark in applications such as colorimetric sensing, smart wearable fabrics and devices, advanced color filters, novel 3D printing, optical cloaking and camouflage, and many others.

Cholesteric liquid crystal photonic actuators (CLCPAs) present remarkable capabilities in structural color response and optical modulation. However, despite their significant advancements, several challenges and limitations remain, posing hurdles to their widespread adoption and further development. Although CLCPAs can exhibit vibrant color changes in response to multiple stimuli, the complex multilayer structure often takes a relatively long time to produce transitions between different color states. As a result, CLCPAs may not be suitable for applications requiring rapid color modulation, such as high-speed displays or fast-acting optical devices. The multilayer structure of CLCPAs lacks the overall stability of the elastomer and may exhibit limited actuation stability and durability over time, particularly when prolonged exposure to temperatures above *T*_g_ or stresses sufficient to initiate material fatigue. CLCPAs play a very important role in flexible sensing and smart wearable systems, but the integration requires addressing compatibility issues with existing ports and processors. And these silicon-based computing and processing devices in the system are often not soft materials. As a result, the development of efficient driving and control systems for CLCPAs is essential for enabling their seamless integration into wearable intelligent systems.

Despite all these challenges, ongoing research efforts continue to address these issues and advance the capabilities of CLCPAs. Innovations in material design, fabrication techniques, and device engineering hold the potential to overcome existing limitations and unlock new opportunities for CLCPAs in diverse fields, including displays, sensors, smart textiles, and optical devices. With the exploration of CLC polymer systems, CLCPAs promise to go beyond the boundaries of structural color and open new avenues for dynamic and responsive optics. Optimizing the stimulus responsiveness of CLCs by doping rich functional materials will increase shape memory and adaptive self-repair and achieve faster color switching and dynamic reconfiguration capabilities. Furthermore, the integration of CLCPAs with soft electronics technologies, such as flexible electronics and wearable devices, offers exciting prospects for novel applications. CLCPA-based smart textiles and wearable sensors could revolutionize fields such as healthcare, sports performance monitoring, and augmented reality, providing users with real-time feedback and personalized experiences. In terms of applications, CLCPAs are poised to make significant contributions to various fields, including displays, signage, camouflage technology, and optical communication. The vibrant and customizable color palette offered by CLCPAs can enable the creation of next-generation displays with enhanced color accuracy, brightness, and energy efficiency. Additionally, CLCPA-based camouflage materials could find utility in military operations, wildlife conservation, and architectural design, offering adaptive concealment solutions tailored to specific environments and requirements. Through continued research and innovation, CLCPAs will play a pivotal role in shaping the future of advanced optical technologies and transformative applications.

## Figures and Tables

**Table 1:** Summary of common dynamic covalent bond networks.

Dynamic Covalent Bonding	Stimuli	Mechanism	Actuation	Ref.
Transesterification reactions	Heat	Exchange of ester and alcohol groups in the presence of acid/base catalysts at elevated temperatures	Reversible shape memory	[62,67,68]
Disulfide bond exchange	Heat/UV	Dissociation and reforming in specific environments	Reversible shape memory	[63,69,70]
B−O bond dynamic chemistry	Heat/water	Reversible B-O bonds with self-healing properties and crosslinkable units	Self-healing	[71,72,73]
Thiol-anhydride bond exchange	Light/heat	The ring-opening reaction of anhydrides with thiols	Programmable shape editing	[74,75]
Carbon radical exchange	Force	Under mechanical stimulation, selectively cleaves unstable bonds, exhibiting fluorescence	Force-induced luminescence	[76,77]

**Table 2:** Summary of common photoisomerization chromophores.

Material	Mechanism	Ref.
spiropyran	The C-O bond breaks and opens the ring, causing local molecular rotation	[156,157,158,159]
diarylethenes	Under UV excitation, the compound rotates to form a colored closed loop, which can undergo opposite changes under visible light irradiation.	[160,161,162]
azobenzene	The cis–trans isomerism formed by –N=N–	[163,164,165,166,167,168]
spirooxazine	Two orthogonal planes connected by a spiral carbon atom as the center rotates under ultraviolet light	[173,174]
naphthopyran	C-O bond breaks and opens the ring, extending the planar and conjugated structure	[175,176]

**Table 3:** Summary of humidity/magnetic/aerodynamic response CLC photonic actuators.

Material	Stimuli	Mechanism	Structural Color	Ref.
CLC/PEG	Humidity	Interpenetrating polymer network (IPN)	Green to red	[183]
CLC polymer film	Humidity/temperature	H-bonded supramolecular CLC resulting in swelling/deswelling	Red to green	[185]
BPLC	Alkaline solution	Manipulating the lattice parameters of the nanostructures	459–653 nm	[186]
CLCN/magnetic composites	Humidity/magnetic	Hygroscopic carboxylate salt groups	Green to red	[39]
CLC/PDMS	Aerodynamic	Gas-pressure-controlled CLC deformation produces structural color	680–460 nm	[192]

## References

[1] Viana J.V.D., Vieira C., Duarte R.C., Romero G.Q. (2022). Predator responses to prey camouflage strategies: A meta-analysis. Proc. R. Soc. B-Biol. Sci..

[2] How M.J., Santon M. (2022). Cuttlefish camouflage: Blending in by matching background features. Curr. Biol..

[3] Wuthrich K.L., Nagel A., Swierk L. (2022). Rapid Body Color Change Provides Lizards with Facultative Crypsis in the Eyes of Their Avian Predators. Am. Nat..

[4] Teyssier J., Saenko S.V., van der Marel D., Milinkovitch M.C. (2015). Photonic crystals cause active colour change in chameleons. Nat. Commun..

[5] Saenko S.V., Teyssier J., van der Marel D., Milinkovitch M.C. (2013). Precise colocalization of interacting structural and pigmentary elements generates extensive color pattern variation in Phelsuma lizards. BMC Biol..

[6] Williams T.L., Senft S.L., Yeo J.J., Martín-Martínez F.J., Kuzirian A.M., Martin C.A., DiBona C.W., Chen C.T., Dinneen S.R., Nguyen H.T. (2019). Dynamic pigmentary and structural coloration within cephalopod chromatophore organs. Nat. Commun..

[7] Sköld H.N., Aspengren S., Wallin M. (2013). Rapid color change in fish and amphibians—Function, regulation, and emerging applications. Pigm. Cell Melanoma Res..

[8] Goda M. (2017). Rapid integumental color changes due to novel iridophores in the chameleon sand tilefish *Hoplolatilus chlupatyi*. Pigm. Cell Melanoma Res..

[9] Mitov M. (2017). Cholesteric liquid crystals in living matter. Soft Matter.

[10] Zhang W., Schenning A.P.H.J., Kragt A.J.J., Zhou G., de Haan L.T. (2021). Reversible Thermochromic Photonic Coatings with a Protective Topcoat. ACS Appl. Mater. Interfaces.

[11] Geng Y., Kizhakidathazhath R., Lagerwall J.P.F. (2022). Robust cholesteric liquid crystal elastomer fibres for mechanochromic textiles. Nat. Mater..

[12] Choi S.S., Wang D., Nam S., Jung W. (2022). Electrically reconfigurable optical color filters using heterogeneous chiral liquid crystals. Res. Sq..

[13] Bobrovsky A., Shibaev V., Cigl M., Hamplová V., Bubnov A. (2023). Fast photo- and electro-optical switching of the polymer- stabilised cholesteric liquid crystal composite prepared by the template method. Liq. Cryst..

[14] Zhang Z.H., Yang X.Y., Zhao Y.J., Ye F.F., Shang L.R. (2023). Liquid Crystal Materials for Biomedical Applications. Adv. Mater..

[15] Hussain S., Zourob M. (2024). Solid-State Cholesteric Liquid Crystals as an Emerging Platform for the Development of Optical Photonic Sensors. Small.

[16] Huang C.T., Chiu C.J., Liu C.K., Cheng K.T. (2024). Method for examining the structural information of heliconical structures of chiral nematics via analyses of the observations of colors and gray levels. Opt. Laser Technol..

[17] Xiang J., Shiyanovskii S.V., Imrie C.T., Lavrentovich O.D. (2014). Electrooptic Response of Chiral Nematic Liquid Crystals with Oblique Helicoidal Director. Phys. Rev. Lett..

[18] Bragg W.H. (1913). The reflection of X-rays by crystals. Nature.

[19] Bragg W.L., Thomson J.J. (1914). The diffraction of short electromagnetic waves by a crystal. Proc. Camb. Philos. Soc..

[20] Dreher R., Meier G. (1973). Optical Properties of Cholesteric Liquid-Crystals. Phys. Rev. A.

[21] Belyakov V.A., Dmitrienko V.E., Orlov V.P. (1979). Optics of Cholesteric Liquid-Crystals. Uspekhi Fiz. Nauk..

[22] Zhou H.M., Wang H., He W.L., Yang Z., Cao H., Wang D., Li Y.Z. (2022). Research Progress of Cholesteric Liquid Crystals with Broadband Reflection. Molecules.

[23] Ma L.L., Li C.Y., Pan J.T., Ji Y.E., Jiang C., Zheng R., Wang Z.Y., Wang Y., Li B.X., Lu Y.Q. (2022). Self-assembled liquid crystal architectures for soft matter photonics. Light-Sci. Appl..

[24] Mitov M. (2012). Cholesteric liquid crystals with a broad light reflection band. Adv. Mater..

[25] Mitov M., Dessaud N. (2006). Going beyond the reflectance limit of cholesteric liquid crystals. Nat. Mater..

[26] Sano H., Ishii A., Mizuno T., Takasago R., Kato S., Ishiguro M., Hayata Y., Nagai M., Ito Y. (2023). Novel cholesteric liquid crystal films creating angle insensitive reflective colors. J. Soc. Inf. Disp..

[27] Choy C.L., Lau K.W.E., Wong Y.W., Yee A.F. (1996). Elastic moduli of a liquid crystalline polymer and its in-situ composites. Polym. Eng. Sci..

[28] Amela-Cortes M., Bruce D.W., Evans K.E., Smith C.W. (2011). Unsymmetric main-chain liquid crystal elastomers with tunable phase behaviour: Elastic response. J. Mater. Chem..

[29] Geng Y., Lagerwall J.P.F. (2023). Multiresponsive Cylindrically Symmetric Cholesteric Liquid Crystal Elastomer Fibers Templated by Tubular Confinement. Adv. Sci..

[30] Wang Y.C., Liu J.Q., Yang S. (2022). Multi-functional liquid crystal elastomer composites. Appl. Phys. Rev..

[31] Park M.S., Kim K., Lee Y.J., Na J.H., Kim S.U. (2023). Deformable Photonic Crystals Based on Chiral Liquid Crystals with Thermal-Mediative Shape Memory Effect. Materials.

[32] Shen W.B., Liu J.S., Du B., Zhuo H.T., Chen S.J. (2021). Thermal- and light-responsive programmable shape-memory behavior of liquid crystalline polyurethanes with pendant photosensitive groups. J. Mater. Chem. A.

[33] Das G., Park S.Y. (2023). Liquid crystalline elastomer actuators with dynamic covalent bonding: Synthesis, alignment, reprogrammability, and self-healing. Curr. Opin. Solid. State Mater. Sci..

[34] Yang X.T., Zhong X., Zhang J.L., Gu J.W. (2021). Intrinsic high thermal conductive liquid crystal epoxy film simultaneously combining with excellent intrinsic self-healing performance. J. Mater. Sci. Technol..

[35] Wang Y.K., Ma Z.H., Li Z.G., Zhang Y.J., Zhang H., Zheng G.L., Xiao Y. (2022). Research on a novel temperature indicating device based on Bragg reflection waveguide of planar texture cholesteric liquid crystal layer. Mol. Cryst. Liq. Cryst..

[36] Kim Y., Mafy N.N., Maisonneuve S., Lin C.Q., Tamaoki N., Xie J. (2020). Glycomacrocycle-Based Azobenzene Derivatives as Chiral Dopants for Photoresponsive Cholesteric Liquid Crystals. ACS Appl. Mater. Interfaces.

[37] Kocakulah G., Koysal O., Kahyaoglu A. (2021). Electro-optical Performance Investigation of Cholesteric Liquid Crystal Containing Azo Dye: Light Shutter Device Application. J. Electron. Mater..

[38] Lan R.C., Shen W.B., Yao W.H., Chen J.Y., Chen X.Y., Yang H. (2023). Bioinspired humidity-responsive liquid crystalline materials: From adaptive soft actuators to visualized sensors and detectors. Mater. Horiz..

[39] Feng W., Pal A., Wang T.L., Ren Z.Y., Yan Y.B., Lu Y.Q., Yang H., Sitti M. (2023). Cholesteric Liquid Crystal Polymeric Coatings for Colorful Artificial Muscles and Motile Humidity Sensor Skin Integrated with Magnetic Composites. Adv. Funct. Mater..

[40] Wang J.W., Cai W.F., He H.L., Cen M.J., Liu J.X., Kong D.L., Luo D., Lu Y.Q., Liu Y.J. (2022). Cholesteric liquid crystal-enabled electrically programmable metasurfaces for simultaneous near- and far-field displays. Nanoscale.

[41] Xiong J.H., Tan G.J., Zhan T., Wu S.T. A scanning waveguide AR display with 100° FOV. Proceedings of the Conference on Optical Architectures for Displays and Sensing in Augmented, Virtual, and Mixed Reality (AR, VR, MR) II, Electr Network.

[42] Yang C.J., Wu B.H., Ruan J., Zhao P., Chen L., Chen D., Ye F.F. (2021). 3D-Printed Biomimetic Systems with Synergetic Color and Shape Responses Based on Oblate Cholesteric Liquid Crystal Droplets. Adv. Mater..

[43] Martinez A.M., McBride M.K., White T.J., Bowman C.N. (2020). Reconfigurable and Spatially Programmable Chameleon Skin-Like Material Utilizing Light Responsive Covalent Adaptable Cholesteric Liquid Crystal Elastomers. Adv. Funct. Mater..

[44] Xu Z., Liu M., Liu Y., Pan Y., Yang L.L., Ge D.T. (2023). Mechano-Optical Response Behavior of Polymer-Dispersed Cholesteric Liquid Crystals for Reversible and Highly Sensitive Force Recorders. ACS Appl. Mater. Interfaces.

[45] Jiang Q., Zhao D.X., Wang J., Yan H.C., Cao S., Qiu Y., Wang H., Liao Y.G., Xie X.L. (2021). Light regulation and long-lived stability of RGB colors in cholesteric liquid crystal physical gels via a mixing strategy. Soft Matter.

[46] Balenko N.V., Shibaev V.P., Bobrovsky A.Y. (2021). Mechano-Optical Response of Novel Polymer Composites Based on Elastic Polyurethane Matrix Filled with Low-Molar-Mass Cholesteric Droplets. Macromol. Mater. Eng..

[47] Yin S.W., Ge S.M., Li X.S., Zhao Y.L., Ma H.M., Sun Y.B. (2023). Recyclable Cholesteric Phase Liquid Crystal Device for Detecting Storage Temperature Failure. ACS Appl. Mater. Interfaces.

[48] Skutnik R.A., Eichler J.C., Mazza M.G., Schoen M. (2021). The temperature dependence of the helical pitch in a cholesteric liquid crystal. Mol. Phys..

[49] Froyen A.A.F., Debije M.G., Schenning A. (2022). Polymer Dispersed Cholesteric Liquid Crystal Mixtures for Optical Time-Temperature Integrators. Adv. Opt. Mater..

[50] Wang K.N., Hu W.T., He W.L., Yang Z., Cao H., Wang D., Li Y.Z. (2024). Research Progress of Electrically Driven Multi-Stable Cholesteric Liquid Crystals. Materials.

[51] Lu H.B., Wang Q., Zhu M.M., Huang P., Xu M., Qiu L.Z., Zhu J. (2022). Electrically controllable reflection bandwidth polymer-stabilized cholesteric liquid crystals with low operating voltage. Liq. Cryst..

[52] Du X.X., Yang F., Liu Y.J., Gleeson H.F., Luo D. (2023). Light-Driven Dynamic Hierarchical Architecture of Three-Dimensional Self-Assembled Cholesteric Liquid Crystal Droplets. Langmuir.

[53] Liu X.J., Qin L., Yu Y.L. (2023). Light-Driven Handedness Inversion of Cholesteric Liquid Crystals. Prog. Chem..

[54] Gevorgyan A.H., Golik S.S. (2022). On the Magnetooptics of Cholesteric Liquid Crystals. Opt. Spectrosc..

[55] Peng Z.L., Huang J.X., Guo Z.G. (2021). Anisotropic Janus materials: From micro-/nanostructures to applications. Nanoscale.

[56] Nam S., Wang D., Lee G., Choi S.S. (2022). Broadband wavelength tuning of electrically stretchable chiral photonic gel. Nanophotonics.

[57] Nam S., Wang D., Kwon C., Han S.H., Choi S.S. (2023). Biomimetic Multicolor-Separating Photonic Skin using Electrically Stretchable Chiral Photonic Elastomers. Adv. Mater..

[58] Kim S.T., Finkelmann H. (2001). Cholesteric Liquid Single-Crystal Elastomers (LSCE) Obtained by the Anisotropic Deswelling Method. Macromol. Rapid Commun..

[59] Cicuta P., Tajbakhsh A.R., Terentjev E.M. (2004). Photonic gaps in cholesteric elastomers under deformation. Phys. Rev. E.

[60] Kizhakidathazhath R., Geng Y., Jampani V.S.R., Charni C., Sharma A., Lagerwall J.P.F. (2019). Facile Anisotropic Deswelling Method for Realizing Large-Area Cholesteric Liquid Crystal Elastomers with Uniform Structural Color and Broad-Range Mechanochromic Response. Adv. Funct. Mater..

[61] Zheng N., Xu Y., Zhao Q., Xie T. (2021). Dynamic Covalent Polymer Networks: A Molecular Platform for Designing Functions beyond Chemical Recycling and Self-Healing. Chem. Rev..

[62] Pei Z.Q., Yang Y., Chen Q.M., Terentjev E.M., Wei Y., Ji Y. (2014). Mouldable liquid-crystalline elastomer actuators with exchangeable covalent bonds. Nat. Mater..

[63] Huang S., Shen Y.K., Bisoyi H.K., Tao Y., Liu Z.C., Wang M., Yang H., Li Q. (2021). Covalent Adaptable Liquid Crystal Networks Enabled by Reversible Ring-Opening Cascades of Cyclic Disulfides. J. Am. Chem. Soc..

[64] Chen L., Bisoyi H.K., Huang Y.L., Huang S., Wang M., Yang H., Li Q. (2021). Healable and Rearrangeable Networks of Liquid Crystal Elastomers Enabled by Diselenide Bonds. Angew. Chem. Int. Ed..

[65] Jiang Z.C., Xiao Y.Y., Yin L., Han L., Zhao Y. (2020). “Self-Lockable” Liquid Crystalline Diels-Alder Dynamic Network Actuators with Room Temperature Programmability and Solution Reprocessability. Angew. Chem. Int. Ed..

[66] McBride M.K., Martinez A.M., Cox L., Alim M., Childress K., Beiswinger M., Podgorski M., Worrell B.T., Killgore J., Bowman C.N. (2018). A readily programmable, fully reversible shape-switching material. Sci. Adv..

[67] Changmai B., Vanlalveni C., Ingle A.P., Bhagat R., Rokhum L. (2020). Widely used catalysts in biodiesel production: A review. RSC Adv..

[68] Yang Y., Terentjev E.M., Zhang Y.B., Chen Q.M., Zhao Y., Wei Y., Ji Y. (2019). Reprocessable Thermoset Soft Actuators. Angew. Chem. Int. Ed..

[69] Wang Z.J., Tian H.M., He Q.G., Cai S.Q. (2017). Reprogrammable, Reprocessible, and Self-Healable Liquid Crystal Elastomer with Exchangeable Disulfide Bonds. ACS Appl. Mater. Interfaces.

[70] Wang Z.J., He Q.G., Wang Y., Cai S.Q. (2019). Programmable actuation of liquid crystal elastomers via “living’’ exchange reaction. Soft Matter.

[71] Ma J., Yang Y., Valenzuela C., Zhang X., Wang L., Feng W. (2022). Mechanochromic, Shape-Programmable and Self-Healable Cholesteric Liquid Crystal Elastomers Enabled by Dynamic Covalent Boronic Ester Bonds. Angew. Chem. Int. Ed..

[72] Lai J.C., Mei J.F., Jia X.Y., Li C.H., You X.Z., Bao Z.A. (2016). A Stiff and Healable Polymer Based on Dynamic-Covalent Boroxine Bonds. Adv. Mater..

[73] Chen L., Chu D., Cheng Z.A., Wang M., Huang S. (2020). Designing seamless-welded liquid-crystalline soft actuators with a “glue-free” method by dynamic boroxines. Polymer.

[74] Kuenstler A.S., Hayward R.C. (2019). Light-induced shape morphing of thin films. Curr. Opin. Colloid Interface Sci..

[75] Dong L.Q., Feng Y.Y., Wang L., Feng W. (2018). Azobenzene-based solar thermal fuels: Design, properties, and applications. Chem. Soc. Rev..

[76] van Galen M., Kaniraj J.P., Albada B., Sprakel J. (2022). Single-Molecule Force Spectroscopy of a Tetraaryl Succinonitrile Mechanophore. J. Phys. Chem. C.

[77] Liu Z.C., Bisoyi H.K., Huang Y.L., Wang M., Yang H., Li Q. (2022). Thermo- and Mechanochromic Camouflage and Self-Healing in Biomimetic Soft Actuators Based on Liquid Crystal Elastomers. Angew. Chem. Int. Ed..

[78] Bapat A.P., Sumerlin B.S., Sutti A. (2020). Bulk network polymers with dynamic B–O bonds: Healable and reprocessable materials. Mater. Horiz..

[79] White T.J., Broer D.J. (2015). Programmable and adaptive mechanics with liquid crystal polymer networks and elastomers. Nat. Mater..

[80] Lu L., Chen X., Liu W., Li H., Li Y., Yang Y. (2023). Facile fabrication of patternable and large-area elastic liquid crystal polymer films. Liq. Cryst..

[81] Zhang P., Zhou G.F., de Haan L.T., Schenning A. (2021). 4D Chiral Photonic Actuators with Switchable Hyper-Reflectivity. Adv. Funct. Mater..

[82] Belmonte A., da Cunha M.P., Nickmans K., Schenning A. (2020). Brush-Paintable, Temperature and Light Responsive Triple Shape-Memory Photonic Coatings Based on Micrometer-Sized Cholesteric Liquid Crystal Polymer Particles. Adv. Opt. Mater..

[83] Kragt A.J.J., Broer D.J., Schenning A.P.H.J. (2018). Easily Processable and Programmable Responsive Semi-Interpenetrating Liquid Crystalline Polymer Network Coatings with Changing Reflectivities and Surface Topographies. Adv. Funct. Mater..

[84] Sun D., Zheng L., Xu X., Du K., An Z., Zhou X., Chen L., Zhu J., Chen D. (2023). Multi-functional stimuli-responsive biomimetic flower assembled from CLCE and MOF-based pedals. Chin. Chem. Lett..

[85] Hatamie A., Angizi S., Kumar S., Pandey C.M., Simchi A., Willander M., Malhotra B.D. (2020). Review—Textile Based Chemical and Physical Sensors for Healthcare Monitoring. J. Electrochem. Soc..

[86] Jung M., Jeon S., Bae J. (2018). Scalable and facile synthesis of stretchable thermoelectric fabric for wearable self-powered temperature sensors. RSC Adv..

[87] Liu X.Y., Lillehoj P.B. (2016). Embroidered electrochemical sensors for biomolecular detection. Lab. Chip..

[88] Li B.T., Xiao G., Liu F., Qiao Y., Li C.M., Lu Z.S. (2018). A flexible humidity sensor based on silk fabrics for human respiration monitoring. J. Mater. Chem. C.

[89] Parrilla M., Cánovas R., Jeerapan I., Andrade F.J., Wang J. (2016). A Textile-Based Stretchable Multi-Ion Potentiometric Sensor. Adv. Healthc. Mater..

[90] Kim S.Y., Jee E., Kim J.S., Kim D.H. (2017). Conformable and ionic textiles using sheath-core carbon nanotube microyarns for highly sensitive and reliable pressure sensors. RSC Adv..

[91] Foelen Y., van der Heijden D.A.C., Verdurmen A.M.J., Mulder D.J., Lub J., Schenning A. (2022). Thermal Paper and Time Temperature Integrators Made From a Structural Colored Polymer Crosslinked With Hydrogen Bonded Cyclohexanoic Acid Derivatives. Adv. Opt. Mater..

[92] Frka-Petesic B., Parton T.G., Honorato-Rios C., Narkevicius A., Ballu K., Shen Q.C., Lu Z.H., Ogawa Y., Haataja J.S., Droguet B.E. (2023). Structural Color from Cellulose Nanocrystals or Chitin Nanocrystals: Self-Assembly, Optics, and Applications. Chem. Rev..

[93] Wang C.X., Tang C.M., Wang Y.F., Shen Y.H., Qi W., Zhang T., Su R.X., He Z.M. (2022). Chiral photonic materials self-assembled by cellulose nanocrystals. Curr. Opin. Solid State Mater. Sci..

[94] Lagerwall J.P.F., Schutz C., Salajkova M., Noh J., Park J.H., Scalia G., Bergstrom L. (2014). Cellulose nanocrystal-based materials: From liquid crystal self-assembly and glass formation to multifunctional thin films. NPG Asia Mater..

[95] Mu X.Y., Gray D. (2015). Formation of chiral nematic films from cellulose nanocrystal suspensions is a two-stage process. Abstr. Pap. Am. Chem. Soc..

[96] Beck S., Bouchard J., Berry R. (2011). Controlling the Reflection Wavelength of Iridescent Solid Films of Nanocrystalline Cellulose. Biomacromolecules.

[97] Frka-Petesic B., Radavidson H., Jean B., Heux L. (2017). Dynamically Controlled Iridescence of Cholesteric Cellulose Nanocrystal Suspensions Using Electric Fields. Adv. Mater..

[98] Frka-Petesic B., Guidetti G., Kamita G., Vignolini S. (2017). Controlling the Photonic Properties of Cholesteric Cellulose Nanocrystal Films with Magnets. Adv. Mater..

[99] Wen X., Zhang J., Li J., Li Y., Shi Y., Lu X., Yang S., Yu J. (2023). Bio-Inspired Cholesteric Phase Cellulose Composite with Thermochromic and Circularly Polarized Structural Color for Multilevel Encryption. Adv. Funct. Mater..

[100] Kim H., Choi J., Kim K.K., Won P., Hong S., Ko S.H. (2021). Biomimetic chameleon soft robot with artificial crypsis and disruptive coloration skin. Nat. Commun..

[101] Schlafmann K.R., Alahmed M.S., Lewis K.L., White T.J. (2023). Large Range Thermochromism in Liquid Crystalline Elastomers Prepared with Intra-Mesogenic Supramolecular Bonds. Adv. Funct. Mater..

[102] van Heeswijk E.P.A., Yang L., Grossiord N., Schenning A.P.H.J. (2019). Tunable Photonic Materials via Monitoring Step-Growth Polymerization Kinetics by Structural Colors. Adv. Funct. Mater..

[103] Chilaya G.S. (2000). Effect of various external factors and pretransitional phenomena on structural transformations in cholesteric liquid crystals. Crystallogr. Rep..

[104] Ranjkesh A., Yoon T.H. (2019). Fabrication of a Single-Substrate Flexible Thermoresponsive Cholesteric Liquid-Crystal Film with Wavelength Tunability. ACS Appl. Mater. Interfaces.

[105] Khandelwal H., van Heeswijk E.P.A., Schenning A., Debije M.G. (2019). Paintable temperature-responsive cholesteric liquid crystal reflectors encapsulated on a single flexible polymer substrate. J. Mater. Chem. C.

[106] van Heeswijk E.P.A., Meerman T., de Heer J., Grossiord N., Schenning A. (2019). Paintable Encapsulated Body-Temperature-Responsive photonic Reflectors with Arbitrary Shapes. ACS Appl. Polym. Mater..

[107] Zhang W.X., Lub J., Schenning A., Zhou G.F., de Haan L.T. (2020). Polymer Stabilized Cholesteric Liquid Crystal Siloxane for Temperature-Responsive Photonic Coatings. Int. J. Mol. Sci..

[108] Froyen A.A.F., Grossiord N., de Heer J., Meerman T., Yang L., Lub J., Schenning A. (2022). Ink-Deposited Transparent Electrochromic Structural Colored Foils. ACS Appl. Mater. Interfaces.

[109] Froyen A.A.F., Schenning A.P.H.J. (2023). A multifunctional structural coloured electronic skin monitoring body motion and temperature. Soft Matter.

[110] Zhang P., Debije M.G., de Haan L.T., Schenning A.P.H.J. (2022). Pigmented Structural Color Actuators Fueled by Near-Infrared Light. ACS Appl. Mater. Interfaces.

[111] Yang J., Zhang X., Zhang X., Wang L., Feng W., Li Q. (2021). Beyond the Visible: Bioinspired Infrared Adaptive Materials. Adv. Mater..

[112] Liu B.H., Yuan C.L., Hu H.L., Sun P.Z., Yu L.H., Zheng Z.G. (2022). Programming multicolour micro-patterns via regional polymer-stabilized heliconical soft architecture. J. Mater. Chem. C.

[113] Radka B.P., Lee K.M., Godman N.P., White T.J. (2022). Electro-optic characteristics of stabilized cholesteric liquid crystals with non-liquid crystalline polymer networks. Soft. Matter..

[114] Lee K.M., Marsh Z.M., Crenshaw E.P., Tohgha U.N., Ambulo C.P., Wolf S.M., Carothers K.J., Limburg H.N., McConney M.E., Godman N.P. (2023). Recent Advances in Electro-Optic Response of Polymer-Stabilized Cholesteric Liquid Crystals. Materials.

[115] Kocakulah G., Koysal O. (2022). Electro-Optical, Dielectric and Morphological Properties of a Cholesteric Liquid Crystal Light Shutter: The Influence of Azo Dye and Quantum Dot Nanoparticles. J. Electron. Mater..

[116] Wang D., Li Y.L., Chu F., Li N.N., Li Z.S., Lee S.D., Nie Z.Q., Liu C., Wang Q.H. (2024). Color liquid crystal grating based color holographic 3D display system with large viewing angle. Light-Sci. Appl..

[117] Osiecka-Drewniak N., Piwowarczyk M., Drzewicz A., Juszynska-Galazka E. (2023). Liquid crystal textures, neural networks and art. Liq. Cryst..

[118] Muraveva V., Kozmík V., Kohout M., Manko A., Piryazev A., Ivanov D., Abramchuk S., Cigl M., Bobrovsky A. (2022). The smectogenity as a crucial factor of broadening of the selective light reflection peak in cholesteric photopolymerizable mixtures. Liq. Cryst..

[119] Lavrentovich O.D. (2020). Electromagnetically tunable cholesterics with oblique helicoidal structure Invited. Opt. Mater. Express.

[120] Salili S.M., Xiang J., Wang H., Li Q., Paterson D.A., Storey J.M.D., Imrie C.T., Lavrentovich O.D., Sprunt S.N., Gleeson J.T. (2016). Magnetically tunable selective reflection of light by heliconical cholesterics. Phys. Rev. E.

[121] De Gennes P.G. (1968). Calcul de la distorsion d’une structure cholesterique par un champ magnetique. Solid State Commun..

[122] Meyer R.B. (1968). Effects of Electric and Magnetic Fields on the Structure of cholesteric Liquid Crystals. Appl. Phys. Lett..

[123] Chornous V., Grozav A., Vovk M., Bratova D., Kasian N., Lisetski L., Gvozdovskyy I. (2023). Oblique helicoidal state of the twist-bend nematic doped by chiral azo-compound. arXiv.

[124] Xiang J., Varanytsia A., Minkowski F., Paterson D.A., Storey J.M.D., Imrie C.T., Lavrentovich O.D., Palffy-Muhoray P. (2016). Electrically tunable laser based on oblique heliconical cholesteric liquid crystal. Proc. Natl. Acad. Sci. USA.

[125] Iadlovska O.S., Maxwell G.R., Babakhanova G., Mehl G.H., Welch C., Shiyanovskii S.V., Lavrentovich O.D. (2018). Tuning selective reflection of light by surface anchoring in cholesteric cells with oblique helicoidal structures. Opt. Lett..

[126] Mrukiewicz M., Cigl M., Perkowski P., Karcz J., Hamplová V., Bubnov A. (2024). Dual tunability of selective reflection by light and electric field for self-organizing materials. J. Mol. Liq..

[127] Liu B.H., Yuan C.L., Hu H.L., Wang H., Zhu Y.W., Sun P.Z., Li Z.Y., Zheng Z.G., Li Q. (2022). Dynamically actuated soft heliconical architecture via frequency of electric fields. Nat. Commun..

[128] Wang H., Totaro M., Beccai L. (2018). Toward Perceptive Soft Robots: Progress and Challenges. Adv. Sci..

[129] Zhao Y.H., Wen L. (2023). Dynamic modeling with quantifying dissipated power density and experimental validation of dielectric elastomer actuators. Smart Mater. Struct..

[130] Chen X., Zhou F., Li G., Cao X., Li T. (2022). Self-powered soft robot in the Mariana Trench. Chin. Sci. Bull..

[131] Tang C., Du B.Y., Jiang S.W., Shao Q., Dong X.G., Liu X.J., Zhao H.C. (2022). A pipeline inspection robot for navigating tubular environments in the sub-centimeter scale. Sci. Rob..

[132] Lee D.Y., Jeong S.H., Cohen A.J., Vogt D.M., Kollosche M., Lansberry G., Menguc Y., Israr A., Clarke D.R., Wood R.J. (2022). A Wearable Textile-Embedded Dielectric Elastomer Actuator Haptic Display. Soft Rob..

[133] Zhao H.C., Hussain A.M., Israr A., Vogt D.M., Duduta M., Clarke D.R., Wood R.J. (2020). A Wearable Soft Haptic Communicator Based on Dielectric Elastomer Actuators. Soft Rob..

[134] Zhao Y.H., Guo Q.W., Wu S., Meng G., Zhang W.M. (2019). Design and experimental validation of an annular dielectric elastomer actuator for active vibration isolation. Mech. Syst. Sig. Process..

[135] Zhao Y.H., Meng G. (2020). A bio-inspired semi-active vibration isolator with variable-stiffness dielectric elastomer: Design and modeling. J. Sound Vib..

[136] Shang Y., Liu J., Guan B., Guo J., Ikeda T., Wang J., Jiang L. (2023). Photo/heat/electricity/pressure-driven photonic pattern with multimode memory effect. Chem. Eng. J..

[137] Kwon C., Nam S., Han S.H., Choi S.S. (2023). Optical Characteristics of Stretchable Chiral Liquid Crystal Elastomer under Multiaxial Stretching. Adv. Funct. Mater..

[138] Zhang W., Tian H., Liu T., Liu H., Zhao F., Li X., Wang C., Chen X., Shao J. (2023). Chameleon-inspired active tunable structural color based on smart skin with multi-functions of structural color, sensing and actuation. Mater. Horiz..

[139] Xu X.Y., Tan M.X., Corcoran B., Wu J.Y., Boes A., Nguyen T.G., Chu S.T., Little B.E., Hicks D.G., Morandotti R. (2021). 11 TOPS photonic convolutional accelerator for optical neural networks. Nature.

[140] Nava G., Ciciulla F., Iadlovska O.S., Lavrentovich O.D., Simoni F., Lucchetti L. (2020). Pitch tuning induced by optical torque in heliconical cholesteric liquid crystals. arXiv.

[141] Jiang Z.C., Xiao Y.Y., Cheng R.D., Hou J.B., Zhao Y. (2021). Dynamic Liquid Crystalline Networks for Twisted Fiber and Spring Actuators Capable of Fast Light-Driven Movement with Enhanced Environment Adaptability. Chem. Mater..

[142] Pulford M., Hatef A. (2024). Study of the photothermal response of a multilayer structure doped with VO_2_@Au nanoshells. Phys. Scr..

[143] Skillin N.P., Bauman G.E., Kirkpatrick B.E., McCracken J.M., Park K., Vaia R.A., Anseth K.S., White T.J. (2024). Photothermal Actuation of Thick 3D-Printed Liquid Crystalline Elastomer Nanocomposites. Adv. Mater..

[144] Wang Z.L., Zhao S.Y., Xiao S.S., Liu S.P., Yao R.Q., Li Y.Q., Wang Y.H., Li Y.G., Tan H.Q. (2023). Heteropoly Blue/Carbon Nanotubes Nanocomposites as High-Performance Photothermal Conversion Materials. Chem. A Eur. J..

[145] Zhang J.Q., Li M., Tan X.J., Shi L., Xie K., Zhao X.L., Wang S.J., Zhao S.Y., Zhang H.Y., Duan X.G. (2023). Confined FeNi alloy nanoparticles in carbon nanotubes for photothermal oxidative dehydrogenation of ethane by carbon dioxide. Appl. Catal. B-Environ. Energy.

[146] Jia G.Z., Li Y., Wu L., Lin Y.X. (2023). High photothermal response rate by synergistic effect of CNT-TA-Ag composite structure. J. Phys. Chem. Solids.

[147] Povolotskiy A.V., Smirnova O.S., Soldatova D.A., Povolotckaia A.V., Lukyanov D.A. (2023). High-Precision Optical Excited Heaters Based on Au Nanoparticles and Water-Soluble Porphyrin. Metals.

[148] Yang Y., Long X., Zhang F.Y., Yan H.Y., Li G.W., Luoshan M.D., Huang C.Y., Zhou L. (2023). Highly Uniform AuPt Bimetallic Nanoplates and Nanorings with Tunable Optical Properties and Enhanced Photothermal Conversion Performance in NIR-II Window. Plasmonics.

[149] Tao X.S., Wu S.Y., Ma Y.Y., Li X.Y., Sun H.Y., Wang Y., Zheng Y.Q. (2023). Gold-based nanostructures for efficient NIR-II photothermal conversion: Hybridizing nanoplates with solid/hollow nanospheres. CrystEngComm.

[150] Chang C.K., Huang T.H. (2023). Enhancement of photothermal effect using a hierarchical plasmonic structure. Appl. Surf. Sci..

[151] Zhang X.L., Luo D.J., Liu Y.K., Wang X., Hu H.L., Ye J.H., Wang D.F. (2023). Efficient photothermal alcohol dehydration over a plasmonic W18O49 nanostructure under visible-to-near-infrared irradiation. J. Photochem. Photobiol. A-Chem..

[152] Luo W.X., Zou M.M., Luo L.X., Ma Y., Chen W.J., Hu X.W., Li Q.L., Jiang X.X. (2024). Efficient enhancement of photothermal conversion of polymer-coated phase change materials based on reduced graphene oxide and polyethylene glycol. J. Energy Storage.

[153] Zheng J.R., Yuan C.S., Ie I.R., Shen H.Z. (2023). S-scheme heterojunction CeO_2_/TiO_2_ modified by reduced graphene oxide (rGO) as charge transfer route for integrated photothermal catalytic oxidation of Hg0. Fuel.

[154] Melo B.L., Lima-Sousa R., Alves C.G., Correia I.J., de Melo-Diogo D. (2023). Sulfobetaine methacrylate-coated reduced graphene oxide-IR780 hybrid nanosystems for effective cancer photothermal-photodynamic therapy. Int. J. Pharm..

[155] Pan X., Grossiord N., Sol J.A.H.P., Debije M.G., Schenning A.P.H.J. (2021). 3D Anisotropic Polyethylene as Light-Responsive Grippers and Surfing Divers. Adv. Funct. Mater..

[156] Guan X.Y., Zhang B.Y., Zhu Y.X., Zheng S., Li D.P., Liu S.Y., Han Q.X. (2023). Fascinating Pathway to Facilitate the Photoisomerization of Spiropyran-Based Nanocomposites. ACS Appl. Mater. Interfaces.

[157] Chen G.N., Lin R.Y., Lei Y.S., Cai P., Huang Y.F., Zhang H.F. (2023). Thiol-ene chemistry incorporates a new spiropyran-containing polyurethane ionogel with photochromic, photomechanical and photoconductive properties. Soft. Matter..

[158] Kumar A., Sahoo P.R., Kathuria I., Prakash K., Kumar S. (2023). Oxazine as an efficient precursor for the development of photochromic spiropyrans. J. Photochem. Photobiol. A-Chem..

[159] Wang X., Xu B., Tian W. (2023). A new function of photochromic spiropyran: An efficient photoinitiator for two-photon polymerization. Light:Sci. Appl..

[160] Podshibyakin V.A., Shepelenko E.N., Dubonosova I.V., Karlutova O.Y., Dubonosov A.D., Bren V.A. (2023). Photo- and Ionochromic Diarylethenes with Receptor Fragments in the Thiazole Bridge. Russ. J. Gen. Chem..

[161] Lvov A.G., Kouame E.K., Khusniyarov M.M. (2023). Light-Induced Dyotropic Rearrangement of Diarylethenes: Scope, Mechanism, and Prospects. Chem. A Eur. J..

[162] Wu N.M.W., Fung T.H.C., Ng M., Yam V.W.W. (2023). Benzo *b* Germole-Fused Diarylethenes as Photochromic Organogermanium Compounds. ACS Mater. Lett..

[163] Itoh T., Kimoto M., Kuroda N., Ishizaki K., Yukihiro E., Shimomoto H., Ihara E. (2023). Light-Responsive Crosslinked Polymer Particles from Heterogeneous Polymerization of an Asymmetric Divinyl Azobenzene Monomer. ACS Appl. Polym. Mater..

[164] Li Y.R., Xue B., Yang J.H., Jiang J.L., Liu J., Zhou Y.Y., Zhang J.S., Wu M.J., Yuan Y., Zhu Z.S. (2024). Azobenzene as a photoswitchable mechanophore. Nat. Chem..

[165] Yi J., Yang Y.J., Song X.M., Zhang Y.X. (2024). Photoinduced deformation behavior of poly(aryl ether)s with different azobenzene groups in the side chain. RSC Adv..

[166] Liu J.C., Han Z.T., Wu P.P., Shang Y.Y., Chen J.S., Jia P. (2023). Photochromic Azobenzene Inverse Opal Film toward Dynamic Anti-Fake Pattern. Molecules.

[167] Kondo M., Nakamura K., Sasai H., Takizawa S. (2023). Azobenzene-based Chiral Photoswitchable Catalysts. J. Synth. Org. Chem. Jpn..

[168] Liu H., Zhang L., Zhang G.Q., Du Q.Y., Wang K., Luo X.L., Wu Z.T. (2023). Molecular design of azobenzene-containing photoresponsive phase change materials with energy storage and photolithography potentials. Dye. Pigm..

[169] Kozlenko A.S., Ozhogin I.V., Pugachev A.D., Lukyanova M.B., El-Sewify I.M., Lukyanov B.S. (2023). A Modern Look at Spiropyrans: From Single Molecules to Smart Materials. Top. Curr. Chem..

[170] Thapa K., Iadlovska O.S., Bisoyi H.K., Paterson D.A., Storey J.M.D., Imrie C.T., Li Q., Shiyanovskii S.V., Lavrentovich O.D. (2021). Combined electric and photocontrol of selective light reflection at an oblique helicoidal cholesteric liquid crystal doped with azoxybenzene derivative. Phys. Rev. E.

[171] Kiryutin A.S., Kozinenko V.P., Yurkovskaya A.V. (2024). Photo-SABRE: Nuclear Spin Hyperpolarization of *cis-trans* Photoswitchable Molecules by Parahydrogen. Chemphotochem.

[172] Skacej G., Querciagrossa L., Zannoni C. (2023). On the Effects of Different *trans* and *cis* Populations in Azobenzene Liquid Crystal Elastomers: A Monte Carlo Investigation. ACS Appl. Polym. Mater..

[173] Zou Y.L., Gao H., Su C.D., Wang M., Gao J. (2024). Photo- and pH-dually responsive hydrogel containing spirooxazine groups. J. Polym. Res..

[174] Zhang T.Z., Lou X.Y., Li X.Y., Tu X., Han J., Zhao B., Yang Y.W. (2023). Tunable Photochromism of Spirooxazine in the Solid State: A New Design Strategy Based on the Hypochromic Effect. Adv. Mater..

[175] Sun Y., McFadden M.E., Osler S.K., Barber R.W., Robb M.J. (2023). Anomalous photochromism and mechanochromism of a linear naphthopyran enabled by a polarizing dialkylamine substituent. Chem. Sci..

[176] Zhang W.A., Cheng Y., Wu M.H., Xie X.J. (2024). Photoswitchable chemical sensing based on the colorimetric pH response of ring-opened naphthopyrans. Sens. Actuators B-Chem..

[177] Qin L., Gu W., Wei J., Yu Y. (2017). Piecewise Phototuning of Self-Organized Helical Superstructures. Adv. Mater..

[178] Yu Y.L., Ma Z.Y., Miao X.Y., Cui Y.Y., Song Y.P., Liu S., Fei T., Zhang T. (2024). Humidity sensors based on cross-linked poly(ionic liquid)s for low humidity sensing. Sens. Actuators B-Chem..

[179] Duan Z.H., Zhang M.X., Jiang Y.D., Yuan Z., Tai H.L. (2024). Emerging electrochemical humidity sensors for zero power consumption and self-powered humidity detection: A perspective. J. Mater. Chem. A.

[180] Meng G., Hu H. (2024). Research on Multi-Point Monitoring Data Grid Model and Inversion Positioning Method for Gas Leakage in Oil and Gas Stations. Sustainability.

[181] Shin W., Hong S., Jeong Y., Jung G., Park J., Kim D., Choi K., Shin H., Koo R.H., Kim J.J. (2023). Low-frequency noise in gas sensors: A review. Sens. Actuators B-Chem..

[182] Xia Y., Guo S.H., Yang L., He S.F., Zhou L.X., Wang M.J., Gao J.Y., Hou M., Wang J., Komarneni S. (2023). Enhanced Free-Radical Generation on MoS_2_/Pt by Light and Water Vapor Co-Activation for Selective CO Detection with High Sensitivity. Adv. Mater..

[183] Zhang L., He W.L., Cui Y.F., Zhang Y.Q., Yang Z., Wang D., Cao H., Li Y.Z. (2022). Preparation and properties of water-responsive films with color controllable based on liquid crystal and poly(ethylene glycol) interpenetrating polymer network. Liq. Cryst..

[184] Lv C., Xia H., Shi Q., Wang G., Wang Y.S., Chen Q.D., Zhang Y.L., Liu L.Q., Sun H.B. (2017). Sensitively Humidity-Driven Actuator Based on Photopolymerizable PEG-DA Films. Adv. Mater. Interfaces.

[185] Herzer N., Guneysu H., Davies D.J.D., Yildirim D., Vaccaro A.R., Broer D.J., Bastiaansen C.W.M., Schenning A. (2012). Printable Optical Sensors Based on H-Bonded Supramolecular Cholesteric Liquid Crystal Networks. J. Am. Chem. Soc..

[186] Hu W., Sun J., Wang Q., Zhang L.Y., Yuan X.T., Chen F.W., Li K.X., Miao Z.C., Yang D.K., Yu H.F. (2020). Humidity-Responsive Blue Phase Liquid-Crystalline Film with Reconfigurable and Tailored Visual Signals. Adv. Funct. Mater..

[187] Cao W.Z., Zhao N., Wang Q.X., Li X.L., Yao L.S., Liu Y.J. (2023). Real-time visual humidity response films of interpenetrating polymer network based on cholesteric liquid crystal and poly (acrylic acid). Liq. Cryst..

[188] Liu J., Tai W.J., Wang D.L., Su J., Yu L. (2022). Cholesteric Liquid Crystal Photonic Hydrogel Films Immobilized with Urease Used for the Detection of Hg^2+^. Chemosensors.

[189] Lu H.F., Wang M., Chen X.M., Lin B.P., Yang H. (2019). Interpenetrating Liquid-Crystal Polyurethane/Polyacrylate Elastomer with Ultrastrong Mechanical Property. J. Am. Chem. Soc..

[190] Shi X.Y., Deng Z.X., Zhang P., Wang Y., Zhou G.F., Haan L.T. (2021). Wearable Optical Sensing of Strain and Humidity: A Patterned Dual-Responsive Semi-Interpenetrating Network of a Cholesteric Main-Chain Polymer and a Poly(ampholyte). Adv. Funct. Mater..

[191] Matsuda T., Kawakami R., Namba R., Nakajima T., Gong J.P. (2019). Mechanoresponsive self-growing hydrogels inspired by muscle training. Science.

[192] Kim S.-U., Lee Y.-J., Liu J., Kim D.S., Wang H., Yang S. (2021). Broadband and pixelated camouflage in inflating chiral nematic liquid crystalline elastomers. Nat. Mater..

[193] Khoshtinat S., Carvelli V., Marano C. (2023). Cellulose acetate for a humidity-responsive self-actuator bilayer composite. Cellulose.

[194] Hou T.J., Wang S.F., Li J.M., Wang Y., Zhang Y., Cao L., Xiong S.X., Fan L.L., Gu F. (2023). Optimal humidity-responsive actuators of heterostructured MXene nanosheets/3D-MXene membrane. Smart Mater. Struct..

[195] Wang K.Y., Chang X.L., Diao Y., Li M.D., Liu F., Meng F.B. (2024). Fabrication of liquid crystal Fe_3_O_4_ composites and their magnetorheological properties. Polym. Adv. Technol..

[196] Wei Q.M., Lv P.R., Zhang Y., Zhang J.W., Qin Z.F., de Haan L.T., Chen J.W., Wang D., Xu B.B., Broer D.J. (2022). Facile Stratification-Enabled Emergent Hyper-Reflectivity in Cholesteric Liquid Crystals. ACS Appl. Mater. Interfaces.

[197] Jiang Q., Ruan H., Sun C.C., Wang T., Zhang Y.P., Qiu Y., Wang H., Liao Y.G., Xie X.L. (2023). Two-Color Cholesteric Liquid Crystalline Gels for Reversible Writing and Erasing Information Encryption. Macromol. Rapid Commun..

[198] Zhang J.X., Zhang W.R., Ma H.T., Geng X.D., Zhang H.W., Liu Q.L., Zhu J., Li C.B., Su Y., Zhu N. (2024). Wearable sensors deriving from cationic-induced 2D-2D co-assembled films for nutrient monitoring. Electrochim. Acta.

[199] Romano C., Lo Presti D., Silvestri S., Schena E., Massaroni C. (2024). Flexible Textile Sensors-Based Smart T-Shirt for Respiratory Monitoring: Design, Development, and Preliminary Validation. Sensors.

[200] Zhuang Z.H., Xuan X.W., Li H.J., Jiang D.L., Li M.J. (2024). A wearable antenna sensor based on ePDA/SiO_2_ nanowalls for the detection of lactic acid in sweat. Sens. Actuators B-Chem..

[201] Truong T., Kim J. (2024). A Wearable Strain Sensor Utilizing Shape Memory Polymer/Carbon Nanotube Composites Measuring Respiration Movements. Polymers.

[202] Mostafa M., Agra-Kooijman D.M., Perera K., Adaka A., West J.L., Jákli A. (2023). Colloidal latex/liquid crystal coatings for thermochromic textiles. Colloid Interface Sci. Commun..

[203] Sheng M.F., Wang W.N., Li L., Zhang L.P., Fu S.H. (2021). All-in-one wearable electronics design: Smart electrochromic liquid-crystal-clad fibers without external electrodes. Colloids Surf. A-Physicochem. Eng. Asp..

[204] Jones C., Wortmann F.J., Gleeson H.F., Yeates S.G. (2020). Textile materials inspired by structural colour in nature. RSC Adv..

[205] Erdem A., Eksin E., Senturk H., Yildiz E., Maral M. (2024). Recent developments in wearable biosensors for healthcare and biomedical applications. Trac-Trends Anal. Chem..

[206] Kulkarni M.B., Rajagopal S., Prieto-Simon B., Pogue B.W. (2024). Recent advances in smart wearable sensors for continuous human health monitoring. Talanta.

[207] Pappot H., Steen-Olsen E.B., Hollaender-Mieritz C. (2024). Experiences with Wearable Sensors in Oncology during Treatment: Lessons Learned from Feasibility Research Projects in Denmark. Diagnostics.

[208] Jha R., Mishra P., Kumar S. (2024). Advancements in optical fiber-based wearable sensors for smart health monitoring. Biosens. Bioelectron..

[209] Zhang S.N., Sun C., Zhang J.Y., Qin S.Y., Liu J.L., Ren Y.X., Zhang L.Y., Hu W., Yang H., Yang D.K. (2023). Reversible Information Storage Based on Rhodamine Derivative in Mechanochromic Cholesteric Liquid Crystalline Elastomer. Adv. Funct. Mater..

[210] Fu J.Y., Liu T., Yan T., Pan Z.J. (2024). Transparent core-sheath composite fibers as flexible temperature sensor based on liquid crystal color change for smart sportswear. J. Mol. Liq..

[211] Ma J.Z., Yang Y.Z., Zhang X., Xue P., Valenzuela C., Liu Y., Wang L., Feng W. (2024). Mechanochromic and ionic conductive cholesteric liquid crystal elastomers for biomechanical monitoring and human-machine interaction. Mater. Horiz..

[212] Park G., Choi Y.S., Yun H.S., Yoon D.K. (2020). Fabrication of Bilayer Dichroic Films Using Liquid Crystal Materials for Multiplex Applications. ACS Appl. Mater. Interfaces.

[213] Zhu Z.K., Gao Y., Lu J.G. (2021). Multi-Pitch Liquid Crystal Filters with Single Layer Polymer Template. Polymers.

[214] Miyata M., Nakajima M., Hashimoto T. (2019). High-Sensitivity Color Imaging Using Pixel-Scale Color Splitters Based on Dielectric Metasurfaces. ACS Photonics.

[215] Park W., Park H., Choi Y.S., Yoon D.K. (2022). Optical Rotation-Based Tunable Color Filter Using Chiral Photonic Crystal. Adv. Opt. Mater..

[216] Sun J.S., Marziale J.J., Haselhuhn A.S., Salac D., Chen J.M. (2024). An ICME framework for short fiber reinforced ceramic matrix composites via direct ink writing. Modell. Simul. Mater. Sci. Eng..

[217] Hausladen M.M., Gorbea G.D., Francis L.F., Ellison C.J. (2024). UV-Assisted Direct Ink Writing of Dual-Cure Polyurethanes. ACS Appl. Polym. Mater..

[218] Li H., Bai H.S., Wang Z., Tan Y.H., Tang Y. (2024). Soft bioinspired pneumatic actuator for adaptive grasping based on direct ink writing method. Sens. Actuators A-Phys..

[219] Kopatz J.W., Reinholtz D., Cook A.W., Tappan A.S., Grillet A.M. (2024). Pressure-based process monitoring of direct-ink write material extrusion additive manufacturing. Addit. Manuf..

[220] Xu X.C., Wang H.H., Shen L., Yang Q.L., Yang Y. (2023). Application and evaluation of fused deposition modeling technique in customized medical products. Int. J. Pharm..

[221] Gottschalk N., Bogdahn M., Quodbach J. (2023). 3D printing of amorphous solid dispersions: A comparison of fused deposition modeling and drop-on-powder printing. Int. J. Pharm. X.

[222] Morales M.A., Maranon A., Hernandez C., Michaud V., Porras A. (2023). Colombian Sustainability Perspective on Fused Deposition Modeling Technology: Opportunity to Develop Recycled and Biobased 3D Printing Filaments. Polymers.

[223] Ye G.L., Jiao Y.F., Zhou P., Sun J.X., Zhu L.K., Gong F., Bai J.M., Liu G., Yan M., Zhang R.B. (2023). Preparation of silicon carbide ceramic slurry for stereolithography- based additive manufacturing. Process. Appl. Ceram..

[224] Pu H., Guo Y.H., Cheng Z.C., Chen Z.X., Xiong J., Zhu X.Y., Huang J.G. (2023). Projection Stereolithography 3D-Printed Bio-Polymer with Thermal Assistance. Polymers.

[225] Tabriz A.G., Gonot-Munck Q., Baudoux A., Garg V., Farnish R., Katsamenis O.L., Hui H.W., Boersen N., Roberts S., Jones J. (2023). 3D Printing of Personalised Carvedilol Tablets Using Selective Laser Sintering. Pharmaceutics.

[226] Tonello R., Conradsen K., Pedersen D.B., Frisvad J.R. (2023). Surface Roughness and Grain Size Variation When 3D Printing Polyamide 11 Parts Using Selective Laser Sintering. Polymers.

[227] Bi R., Li X.H., Ou X.C., Huang J.Q., Huang D.T., Chen G.L., Sheng Y., Hong W., Wang Y., Hu W.J. (2024). 3D-Printed Biomimetic Structural Colors. Small.

[228] Choi J., Choi Y., Lee J.H., Kim M.C., Park S., Hyun K., Lee K.M., Yoon T.H., Ahn S.K. (2024). Direct-Ink-Written Cholesteric Liquid Crystal Elastomer with Programmable Mechanochromic Response. Adv. Funct. Mater..

[229] Sol J., Sentjens H., Yang L.T., Grossiord N., Schenning A., Debije M.G. (2021). Anisotropic Iridescence and Polarization Patterns in a Direct Ink Written Chiral Photonic Polymer. Adv. Mater..

[230] Lu X.L., Ambulo C.P., Wang S.T., Rivera-Tarazona L.K., Kim H., Searles K., Ware T.H. (2021). 4D-Printing of Photoswitchable Actuators. Angew. Chem. Int. Ed..

[231] Liu J.Q., Gao Y.C., Wang H.H., Poling-Skutvik R., Osuji C.O., Yang S. (2020). Shaping and Locomotion of Soft Robots Using Filament Actuators Made from Liquid Crystal Elastomer-Carbon Nanotube Composites. Adv. Intell. Syst..

[232] Agrawal A., Chen H.Y., Kim H., Zhu B.H., Adetiba O., Miranda A., Chipara A.C., Ajayan P.M., Jacot J.G., Verduzco R. (2016). Electromechanically Responsive Liquid Crystal Elastomer Nanocomposites for Active Cell Culture. ACS Macro Lett..

[233] Zhang W.Q., Chen J.Z., Li X., Lu Y. (2020). Liquid Metal-Polymer Microlattice Metamaterials with High Fracture Toughness and Damage Recoverability. Small.

[234] Kotikian A., Morales J.M., Lu A., Mueller J., Davidson Z.S., Boley J.W., Lewis J.A. (2021). Innervated, Self-Sensing Liquid Crystal Elastomer Actuators with Closed Loop Control. Adv. Mater..

[235] Liu X.H., Debije M.G., Heuts J.P.A., Schenning A. (2021). Liquid-Crystalline Polymer Particles Prepared by Classical Polymerization Techniques. Chem. A Eur. J..

[236] Cipparrone G., Mazzulla A., Pane A., Hernandez R.J., Bartolino R. (2011). Chiral self-assembled solid microspheres: A novel multifunctional microphotonic device. Adv. Mater..

[237] Belmonte A., Ussembayev Y.Y., Bus T., Nys I., Neyts K., Schenning A. (2020). Dual Light and Temperature Responsive Micrometer-Sized Structural Color Actuators. Small.

[238] Donato M.G., Mazzulla A., Pagliusi P., Magazzù A., Hernandez R.J., Provenzano C., Gucciardi P.G., Maragò O.M., Cipparrone G. (2016). Light-induced rotations of chiral birefringent microparticles in optical tweezers. Sci. Rep..

[239] Ussembayev Y.Y., De Witte N., Liu X.H., Belmonte A., Bus T., Lubach S., Beunis F., Strubbe F., Schenning A., Neyts K. (2023). Uni- and Bidirectional Rotation and Speed Control in Chiral Photonic Micromotors Powered by Light. Small.

[240] Kelley J.L., Kelley L.A., Badcock D.R. (2022). 3D animal camouflage. Trends Ecol. Evol..

[241] Widmann M.E., van Elden S., Meeuwig J.J. (2023). Colour change and colour phases in Lethrinidae with insights into ecology. Ecol. Evol..

[242] Wardill T.J., Feord R.C., Sumner M.E., Pusdekar S., Kalra L., Gonzalez-Bellido P.T. (2020). Binocular stereopsis in cuttlefish improves prey targeting. Integr. Comp. Biol..

[243] Nityananda V., Tarawneh G., Henriksen S., Umeton D., Simmons A., Read J.C.A. (2018). A Novel Form of Stereo Vision in the Praying Mantis. Curr. Biol..

[244] Davey M.P., Srinivasan M.V., Maddess T. (1998). The Craik-O’Brien-Cornsweet illusion in honeybees. Naturwissenschaften.

[245] Adams W.J., Graf E.W., Anderson M. (2019). Disruptive coloration and binocular disparity: Breaking camouflage. Proc. R. Soc. B-Biol. Sci..

[246] Xu C., Zhang B., Guo T.C., Zhu R.P., Xu G.Y. (2023). The relationship between spatial color mixing with color and radiation similarity in digital camouflage study. Infrared Phys. Technol..

[247] Zhang P., de Haan L.T., Debije M.G., Schenning A. (2022). Liquid crystal-based structural color actuators. Light-Sci. Appl..

[248] Sun C., Zhang S., Ren Y., Zhang J., Shen J., Qin S., Hu W., Zhu S., Yang H., Yang D. (2022). Force-Induced Synergetic Pigmentary and Structural Color Change of Liquid Crystalline Elastomer with Nanoparticle-Enhanced Mechanosensitivity. Adv. Sci..

